# Unconventional Magnetization below 25 K in Nitrogen-doped Diamond provides hints for the existence of Superconductivity and Superparamagnetism

**DOI:** 10.1038/s41598-019-45004-6

**Published:** 2019-06-19

**Authors:** J. Barzola-Quiquia, M. Stiller, P. D. Esquinazi, A. Molle, R. Wunderlich, S. Pezzagna, J. Meijer, W. Kossack, S. Buga

**Affiliations:** 1 Division of Superconductivity and Magnetism, Felix Bloch Institute for Solid State Physics, University of Leipzig, 04103 Leipzig, Germany; 2 Division of Applied Quantum System, Felix Bloch Institute for Solid State Physics, University of Leipzig, 04103 Leipzig, Germany; 3 Division of Molecular Physics, Peter Debye Institute for Soft Matter Physics, University of Leipzig, 04103 Leipzig, Germany; 4 Technological Institute for Superhard and Novel Carbon Materials, 7a Centralnaya street, Troitsk, Moscow, 108840 Russia; 5 Moscow Institute of Physics and Technology, 9 Institutskiy per., Dolgoprudny, Moscow Region, 141701 Russia; 6 Present Address: Locally-Sensitive & Time-Resolved Spectroscopy (EM-ALTS); and Institute for Chemistry and Biochemistry, Helmholtz-Zentrum Berlin f. Materialien und Energie GmbH; and Freie Universität Berlin, Hahn-Meitner-Platz 1, 14109 Berlin-Wannsee, Germany

**Keywords:** Superconducting properties and materials, Magnetic properties and materials

## Abstract

The magnetization of nitrogen-doped single crystalline diamond bulk samples shows unconventional field and temperature hysteresis loops at *T* ${\boldsymbol{\lesssim }}$ 25 K. The results suggest the existence of superparamagnetic and superconducting regions in samples with nitrogen concentration <200 ppm. Both phases vanish at temperatures above 25 K where the samples show diamagnetic behavior similar to undoped diamond. The observation of superparamagnetism and superconductivity is attributed to the nitrogen doping and to the existence of defective regions. From particle-induced X-ray emission with ppm resolution we rule out that the main observations below 25 K are due to magnetic impurities. We investigated also the magnetic properties of ferromagnetic/high-temperature superconducting oxide bilayers. The magnetization results obtained from those bilayers show remarkable similarities to the ones in nitrogen-doped diamond.

## Introduction

Diamond, a natural allotrope of carbon, is transparent and insulating, a good thermal conductor at room temperature and the hardest naturally occurring material on earth. Because of its large band gap of about 5.45 eV, intrinsic diamond is a good electrical insulator. However, real crystals may show finite electrical conductivity due to impurities and defects. Doping with boron provides bulk conductivity in a broad range up to a metallic state, depending on the boron content. Hydrogen and lithium also have doping effects in diamond, but these atoms tend to migrate at normal temperature, thus the electrical properties are unstable in these cases. Nitrogen easily incorporates into the diamond lattice and may act as a donor. However, in most cases the nitrogen will form P1 centres or other N-related defects like conglomerates. The ionization energy of nitrogen donor in diamond is high, about 1.7 eV, therefore even heavy nitrogen doping does not induce free electron conductivity in diamond. Only phosphor and arsenic act as stable donor atoms in diamond, but they are not typical substitution atoms in the diamond lattice because of their large difference in ion radius compared to carbon. Moreover, the structure of donors is complicated and still not well understood.

In most cases defects in the diamond lattice can play a key role in electric transport. Defects induce intermediate states within the band gap, and their density and activation energy for carriers may vary in a broad range. Several details of the influence of defects are not well explored yet. Besides substitution states, interstitial impurities also affect the structure of defects and the optical and electrical properties of diamond. A recent review on the subject can be read in^1^.

Before we review the search for superconductivity (SC) and magnetic order or superparamagnetism (SPM) in diamond, we would like to stress that the research of these ordering phenomena in allotrope of carbon and carbon-based materials has a long history. For example, SC was reported in single-walled^2,3^ and multi-walled^4,5^ carbon nanotubes up to a critical temperature of ${T}_{c}\sim 20\,{\rm{K}}$. Hints for the existence of granular SC in bulk as well as in micrometer size flakes of highly oriented pyrolytic and natural graphite samples were reported in the last 20 years. Recent theoretical work^6–9^ as well as new experimental evidence^10,11^ indicate the existence of high temperature SC at certain interfaces of graphite samples.

As it will become clear later, the results presented in this study indicate that both phenomena, i.e. SC and SPM, in diamond are caused by certain doped and/or defective regions in the samples. It is of interest, therefore, to take a look at previous work on “defect-induced” phenomena. Evidence for magnetic order was found in pure graphite, in proton-irradiated graphite, in sulphuric acid treated graphite powder and in a large list of defective graphite samples (for a recent review see^12^). Its origin is related to defects already existing in the samples or produced by ion irradiation. Results obtained by soft X-rays magnetic circular dichroism (XMCD) reveal further that the magnetic order is mainly localized at the valence band^13^. The strong spin polarization of the valence band appears to be a general property in materials showing defect induced magnetism (DIM).

Superconductivity has been found in semiconducting superlattices up to 6 K and attributed to dislocations^14^ at the interfaces^15^. We also note that superconductivity was found at the interfaces of Bi and BiSb bicrystals with ${T}_{c}\lesssim 21\,{\rm{K}}$^16–19^. The possibility to have both ordering phenomena, i.e. high temperature superconductivity and/or magnetic order, at the surface of rhombohedral graphite or at the two-dimensional interfaces between different stacking orders in graphite, has recently also been studied theoretically^6–8,20–22^ and experimentally^23–25^. The SC phenomenon is enhanced due to a dispersionless electron band, a so-called flat band. Strain-induced superconductivity at interfaces of semiconducting layers has also been treated theoretically based on the influence of partial flat-bands^26^.

First works on SC in diamond^27,28^ were reported in boron-doped diamond, synthesized at high pressure, with a transition temperature ${T}_{c}=2.5\ldots 2.8\,{\rm{K}}$. Local scanning tunneling microscopy studies of superconducting boron-doped diamond samples revealed, however, inhomogeneous, granular superconductivity with unclear boron concentration within the superconducting regions^29^. On the other hand, boron-doped carbon nanotubes were reported to show superconductivity below 12 K^30^. Moreover, an apparent coexistence of SC and magnetic order was recently reported in hydrogenated boron-doped nanodiamond films at temperatures below 3 K^31^. In that work the authors suggest that spin fluctuations might be responsible for the Cooper pairing. Note that hydrogenation might trigger local magnetic order in diamond with a Curie temperature above 400 K, similar to the magnetic order found in hydrogen-graphite systems^13,32^. Ferromagnetism was also found in nitrogen implanted nanocrystalline diamond films^33^.

According to the BCS model for superconductivity, superconductivity and magnetic order are phenomena not expected to coexist. A long standing belief in the scientific community before the discovery of superconductivity in iron-based pnictides (such as LaOFFeAs) with antiferromagnetic order^34^. The simultaneous occurrence of superconductivity and magnetism was observed in alloys such as HoMo_6_S_8_^35^ and ErRh_4_B_4_^36^. The coexistence of FM and SC was reported for the hybrid ruthenate-cuprate compound RuSr_2_GdCu_2_O_8_^37^. In the case of ferromagnetic superconductors, such as UGe_2_^38^, URhGe^39^ and ZrZn_2_^40^, SC coexists with FM. Furthermore, a recent theoretical work generalized Eliashberg’s equations of strong coupling superconductivity to systems with flat bands taking into account electron-phonon and electron-electron interactions, i.e. the Stoner magnetism^41^. One of the main results of this work predicts the coexistence of both ordering phenomena in form of metastable domains inside the bulk of the sample. From all this previous work one may conclude that there are chances to trigger both ordering phenomena in doped and/or defective diamond.

In this work we present experimental results on the magnetic properties of a total of eight diamond crystals with different contents of nitrogen. The samples show temperature and magnetic field hysteresis in the magnetization at temperatures $T\lesssim 25\,{\rm{K}}$. The overall results can be well explained by the simultaneous existence of superparamagnetic and superconducting regions. Through our characterization we can rule out that the origin for the SPM is related to magnetic impurities. At temperatures above $T\simeq 25\,{\rm{K}}$ both phenomena vanish, indicating that they should have a common origin. The measured time dependence of the magnetization due to thermally activated flux creep follows a stretched exponential indicating the existence of a dynamic heterogeneity similar to that found in superconducting systems with magnetic phases^42,43^. The results obtained in this study suggest that defective or strained regions trigger both phenomena in the diamond lattice, a lattice composed of only light elements, C and N, this last with a relatively small concentration. As a support to our interpretation of the magnetization hysteresis in terms of a, at least, superposition of both phenomena, magnetization measurements were done in bilayers of ferromagnetic/superconducting samples at fields applied parallel to the main surface of the bilayers.

This paper is organized as follows: In Section 0.1 we present the magnetic field and temperature dependence of the magnetization of the diamond samples (Section 0.1.1), the time dependence (Section 0.1.2) and the contribution of magnetic impurities in the magnetization (Section 0.1.3). In Section 0.2 we present the characterization of the samples with optical microscopy, photoluminescence, electron paramagnetic resonance and infrared spectroscopy. The results of the magnetization field loops measured in FM/SC bilayers (LSMO/YBCO, Ni/YBCO) for fields parallel to the main area of the samples are presented in Section 0.3. In Section 1 we discuss the results obtained from the diamond samples and propose a simple way to understand semiquantitatively the anomalous hysteresis loops taking into account the strong similarities with the field loops measured in the FM/SC bilayers. Conclusion is given in Section 2.

## Results

### Magnetic Moment of N-doped Diamond samples

#### Field and temperature dependence of the magnetization

The magnetic moment *m* of the samples shown in the main text of the article was measured with the large surface areas of the samples parallel to the applied magnetic field. In order to check for the existence of some magnetic (or size) anisotropy we have measured the samples also with the field applied in other directions. The obtained results show no direction dependence within experimental resolution. This fact appears to rule out well defined defective lines observed at the surface of several diamond samples as the source (see Fig. S15(a–d) in the supplementary information (SI)) of the signals measured.

The field hysteresis loops at constant temperature $T < 60\,{\rm{K}}$ were measured as follows: we first cooled the sample from a temperature of 75 K at zero field to the selected temperature. After achieving this temperature the magnetic field was swept from $0\,{\rm{T}}\to +\,7\,{\rm{T}}$, then from $+\,7\,{\rm{T}}\to -\,7\,{\rm{T}}$ and finally back to +7 T. After this procedure, the applied field was set to zero and the sample heated to $T=75\,{\rm{K}}$. Next, the sample was cooled at zero field to a new fixed constant temperature and the same measurement sequence was used.

**Figure 1:**
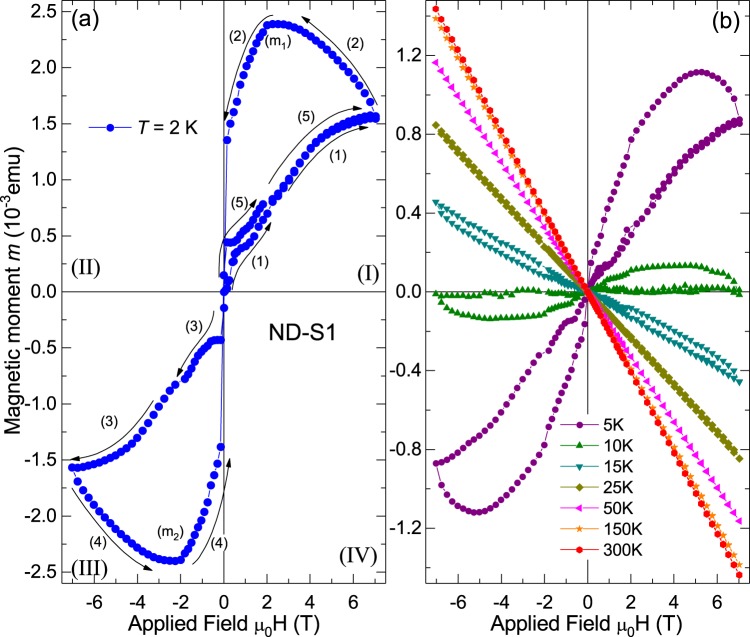
Magnetic moment as a function of the applied field for sample ND-S1 (mass 42.6 mg). (**a**) Results measured at $T=2\,{\rm{K}}$ after subtraction of the diamagnetic background, the numbers and arrows indicate the field sweep step and direction. (**b**) Data obtained at higher temperatures without subtraction.

We discuss first the results of sample ND-S1, which contains $\simeq 86\,{\rm{ppm}}$ of N-atoms. Qualitative similar results were obtained for all N-doped diamond samples. The size of the field hysteresis shown in Fig. 1, as example, depends on the nitrogen content, as will become clear below. In Fig. 1(a) the magnetic moment at $T=2\,{\rm{K}}$ is shown, the numbered arrows indicate the corresponding sequence step explained above. The field hysteresis is completely unusual compared to a ferromagnetic or superconducting hysteresis. The path labeled (1) in quadrant (I) looks similar to a FM case. Path (2) is unusual because the magnetic moment increases to a maximum (m_1_) decreasing the applied field, followed by a decrease and a sudden collapse to approximately zero around zero applied field.

When the field is reversed, see path (3) in Fig. 1(a), the magnetic moment begins at approximately zero and decreases abruptly. This is different from what one would expect for a ferromagnetic or superconducting material, where a crossover from quadrant (I) to (III) would include quadrant (II) as well. Paths (3) and (4) show a similar change of the magnetic moment as in quadrant (I). The final path (5) in quadrant (I) is also unusual because the magnetic moment follows closely path (1), in contrast to what is expected for an usual ferromagnet or superconductor. Similar results were obtained up to $T\simeq 25\,{\rm{K}}$, see Fig. 1(b).

**Figure 2:**
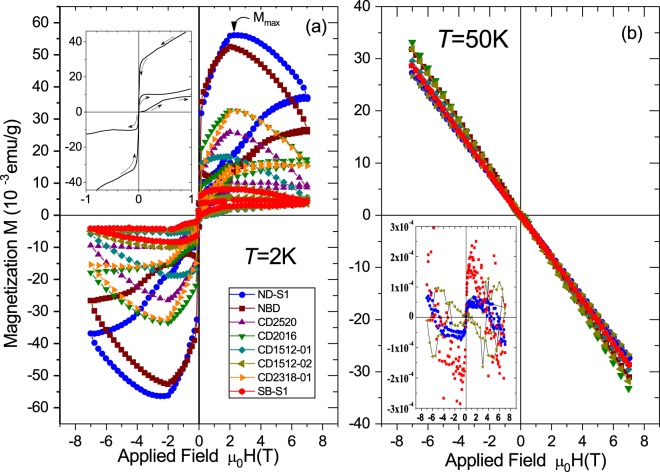
Magnetization vs. applied field using the same field sequence as in Fig. 1(a) for all investigated samples at $T=2\,{\rm{K}}$ after subtraction of a temperature independent linear diamagnetic background. The inset blows out the low field region of the same data of sample ND-S1 shown in the main panel. (**b**) Field dependent magnetization at $T=50\,{\rm{K}}$ of all samples without any subtraction. The inset shows as example the data of three of the samples (SB-S1, ND-S1, CD1512-02) after subtraction of the linear diamagnetic background. Note that the both axes are in the same units as in the main panel. It means that at 50 K the maximum deviation from a linear diamagnetic behavior measured at fields of the order of, e.g., 2 T is at least ~5 orders of magnitude smaller than the signals observed at 2 K, see panel (a).

We have investigated other samples with different nitrogen content (which can be easily recognized from the intensity of the yellow color). The results are presented in Fig. 2(a). To highlight that there is a correlation between the N-content and the unconventional field hysteresis we have plotted in Fig. 2(a) the results normalized to the corresponding sample mass, i.e. the magnetization *M*. The results in Fig. 2(a) are after subtraction of the linear diamagnetic contribution, see Fig. 2(b). The results are similar in all N-doped samples, with the largest field hysteresis corresponding to samples with higher nitrogen concentration. The raw data presented in Fig. 2(b) obtained at $T=50\,{\rm{K}}$ indicate that the presence of nitrogen does not influence the intrinsic diamagnetism of pure diamond because the magnetization data are nearly identical for all samples. Raw data at other temperatures are included in the SI.

**Figure 3:**
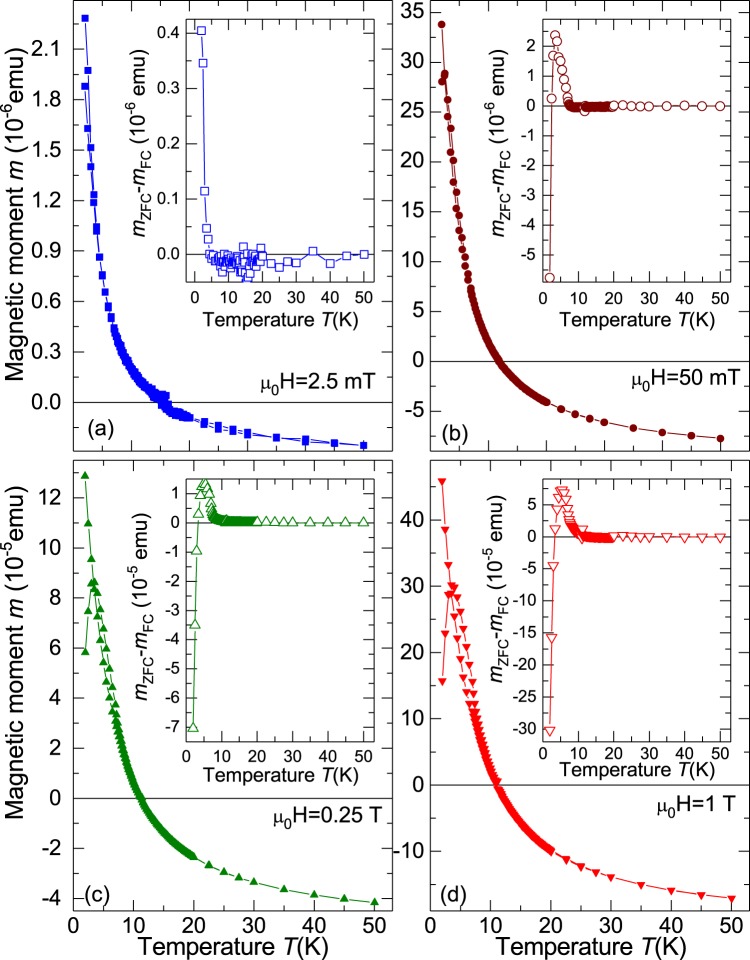
Zero field cooled (ZFC) and field cooled (FC) magnetic moment as a function of temperature at different constant applied fields. The insets in (**a**–**d**) show the difference *m*_*d*_. The data correspond to sample ND-S1.

In the next step, the magnetic moment as a function of temperature at different constant applied magnetic fields was measured. The results of these zero-field-cooled (ZFC) and field-cooled (FC) measurements are presented in Fig. 3. At low temperatures the magnetic moment is positive. At temperatures around $T\approx 12\,{\rm{K}}$, the diamagnetic contribution becomes dominant. In the case of non-doped diamond, the magnetic moment is essentially diamagnetic and basically temperature independent from 2 K to 300 K (see Fig. S1 in the SI).

The difference between the ZFC and FC curves, ${m}_{d}={m}_{{\rm{ZFC}}}(T)-{m}_{{\rm{FC}}}(T)$, is plotted in the insets of Fig. 3. This difference is unconventional compared to pure ferromagnetic or superconducting materials. At low applied fields, see Fig. 3(a), *m*_*d*_ is positive and vanishes at $T\ge 12\,{\rm{K}}$. At higher fields *m*_*d*_ is negative at low temperatures, gets positive at higher temperatures and decreases to zero after a maximum, see Fig. 3(b–d).

**Figure 4:**
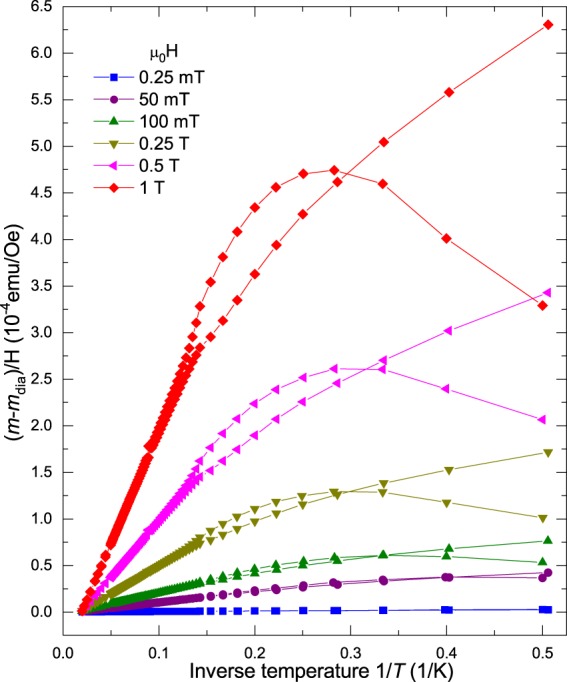
Field susceptibility defined as the measured magnetic moment *m* minus a temperature independent background *m*_*dia*_ divided by the applied field *H* vs. the inverse of temperature. We show the data of sample CD2318-01, as example, in the ZFC and FC states. Note the reversible 1/*T* behavior at high enough temperatures. The irreversible behavior is observed at low enough temperatures for a given applied field.

The decrease of the magnetic moment with temperature at all applied fields shown in Fig. 3(a–d) appears to follow a simple Curie-like paramagnetic behavior. Were this the case, we expect a straight line and a scaling at all applied fields of the susceptibility *χ* vs. inverse temperature 1/*T*. To check this we plot the susceptibility defined as $\chi =(m-{m}_{dia})$/*H* vs. 1/*T*, where *m*_*dia*_ is the diamagnetic background measured at 50 K, see Fig. 2(b). The results presented in Fig. 4 for one of the samples suggest that one of the magnetic contributions to the overall magnetic response of the samples should be superparamagnetic (SPM), not simply paramagnetic. Note the large increase of the susceptibility linear slope at high temperatures with applied field shown in Fig. 4. It means that the applied field magnetizes small magnetic regions of the sample, similar to a PM, but having a much larger susceptibility that strongly increases the lower the temperature. Such SPM behavior has been observed and well studied in a variety of systems like magnetic nano particles, see, e.g.^44^. To simulate the field hysteresis loops obtained in all the N-doped samples, see Section 1, we will assume therefore the existence of a SPM state that shows no hysteresis in field, as is usually the case for this magnetic phase above the blocking temperature^44^.

#### Time dependent magnetization

In general, superconductors show a time dependent magnetization after a field change due to flux creep^45^. Also ferromagnets can show a creep behavior in the magnetization due to the movement of the domain walls after a sudden change of the field. Superparamagnets show creep specially close to the blocking temperature. To check whether the magnetization depends on time, time dependent magnetization measurements at different constant temperatures and applied magnetic fields were performed.

Time-up (TU) measurements were done as follows: the sample was kept at $T=75\,{\rm{K}}$ ($B=0\,{\rm{T}}$), followed by a cool down to the desired temperature, i.e. $T=2\,{\rm{K}}$. A magnetic field was then applied after the set temperature was reached and remained constant within experimental resolution. The magnetic moment was measured for $t\approx 44\,{\rm{\min }}$. Before the measurements were done at a new magnetic field, the sample was heated at $T=75\,{\rm{K}}$. The TU results are shown in 5(a).

**Figure 5:**
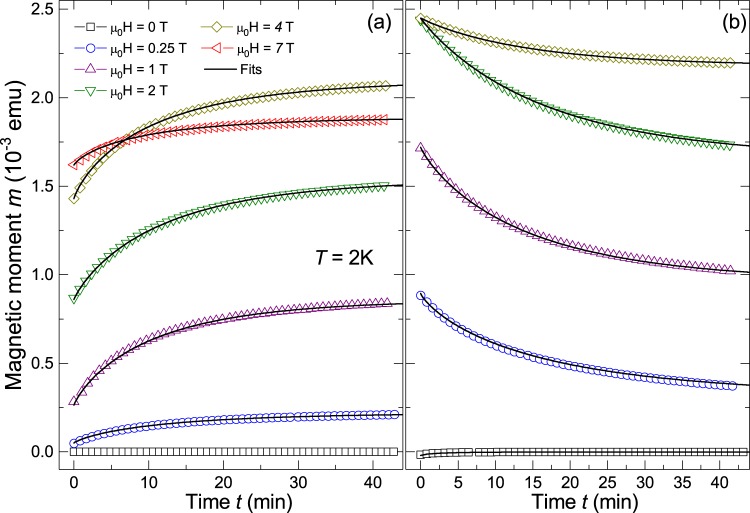
Time dependent magnetic moment measured at different constant fields: (**a**) TU measurements, (**b**) TD measurements. The lines are fits to the stretched exponential function as explained in the text.

Time-down (TD) measurements were done after the TU measurement at $B\mathrm{=7\ }{\rm{T}}$. In this case the magnetic field was set starting always from 7 T to the next desired field and the magnetic moment was measured for $t\approx 44\,{\rm{\min }}$. The obtained results, at $T=2\,{\rm{K}}$ and at different constant fields, are presented in Fig. 5(b). Results at other temperatures are given in the SI (see Fig. S2).

The TU and TD measurements confirm that the magnetic moment has a time dependence, which changes with temperature and vanishes at $T\approx 25\,{\rm{K}}$, see Figs S2 and S3 in the SI. Such behaviour known as flux creep can be described by a model proposed by Anderson and Kim (AK)^46,47^. Although the AK model can describe the results in our case, the necessary fit parameters are physically inconsistent. Instead, a stretched exponential function $m(t)=m(0)-{m}_{1}\,\exp (\,-\,{(t/{t}_{0})}^{\beta })$ describes our data very well over the entire temperature range and applied fields. In this function ${t}_{0}$ is a characteristic relaxation time and $\beta$ a further fit parameter, where $\beta =1$ corresponds to a single exponential relaxation expected for a homogeneous system, and $\beta  < 1$ implies a broad distribution.

In Fig. S3 of the SI we have plotted the corresponding parameters ${t}_{0}$ and $\beta$ as a function of temperature. The characteristic time interval ${t}_{0}$ shows a decrease increasing temperature and vanishes at $T\approx 25\,{\rm{K}}$. On average, the values obtained for $\beta$ are close to ≈0.8 and are nearly temperature independent. A similar value was found in studies on the dynamics of spin glasses above the freezing temperature for small clusters^48^. In the case of $\kappa -{({\rm{ET}})}_{2}{\rm{Cu}}[{\rm{N}}{({\rm{CN}})}_{2}]{\rm{Br}}$, the values were distributed within $0.5 < \beta  < 0.95$^42^ and for LaSrCuO $\beta \approx 0.48$^43^. We note that time dependent measurements at other field directions did not show any difference within experimental resolution, indicating no directional preference, as expected for disordered systems.

The existence of stretched relaxation usually indicates dynamic heterogeneity, a concept used in the study of a wide variety of disordered systems^49^, such as the high *T*_*c*_ superconductor LaSrCuO^43^, where superconductivity and (striped and glassy) magnetic order coexist. For this superconductor NMR measurements of the ^139^La spin-lattice relaxation were reported, which displayed a stretched-exponential time dependence in both pure and disordered single crystals. Proton NMR measurements on the organic superconductor $\kappa -{({\rm{ET}})}_{{\rm{2}}}{\rm{Cu}}[{\rm{N}}{({\rm{CN}})}_{{\rm{2}}}]{\rm{Br}}$^42^ (${T}_{c}=11.6\,{\rm{K}}$) exhibited also stretched exponential spin-lattice relaxation below $T\approx 25\,{\rm{K}}$, compatible with an inhomogeneous magnetic phase that develops in the normal state and coexists with superconductivity. From all that published work we may conclude that the measured time dependence in the N-doped diamond samples suggests the existence of inhomogeneous magnetic phases. As will become clear in the discussion, see Section 1, our data can be understood assuming the existence of a SPM phase together with a superconducting-like signal.

#### Magnetic contribution from magnetic impurities

A paramagnetic or ferromagnetic contribution in nominally non-magnetic materials may originate from magnetic impurities present in the materials. In general and for low concentration of ferromagnetic impurities, their contribution to the total magnetic moment can be observed in the magnetic hysteresis curves only after the subtraction of the linear diamagnetic contribution. In order to discard the possibility that the signals observed below 25 K in the doped diamond crystals are due to magnetic impurities, we have studied the magnetic signals above 25 K and compared them with the expected ones according to the measured impurity concentration. To identify the magnetic impurities and quantify the amount present in the sample, we have carried out particle induced x-rays emission (PIXE) experiments on the sample ND-S1. This non-destructive technique is able to resolve contaminants with parts-per-million (ppm) resolution. For the PIXE analysis we use a focused proton beam with an approximated diameter of 1 *μ*m and an energy of 2.25 MeV. This yields a penetration depth of about 30 *μ*m, calculated with the software SRIM^50^.

**Figure 6:**
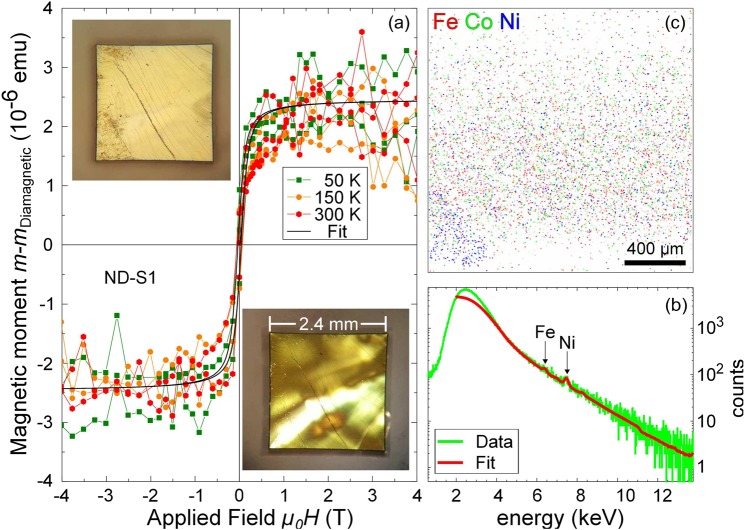
(**a**) Field hysteresis obtained after subtraction of linear diamagnetic contribution at different constant temperatures for the nitrogen doped sample ND-S1. The continuous line is a fit to the model for a ferromagnetic hysteresis from^56,57^. The inset at the upper left is an optical image of the main surface of the sample. The inset at bottom right shows an optical birefringence image of the same sample indicating stress and inhomogeneity. (**b**) Accumulated PIXE spectrum of the whole scan area. The energies corresponding to Fe and Ni impurities are shown. (**c**) Lateral distribution of the PIXE signal of Fe, Co and Ni in a false color picture (obtained from the sample ND-S1).

The main signal of the characteristic X-rays of iron and nickel arises from a region between the sample surface and a depth of about 20 *μ*m. The data analysis was performed using the program GeoPIXE 6.4x^51,52^. The scan region of 1.6 mm × 1.6 mm was located at the centre of the diamond sample. For the quantification of the FM impurity concentrations the element correlated K_*α*_ line intensities were used. The PIXE measurements on sample ND-S1 shown in Fig. 6(b) indicate that the major contamination is due to Fe and Ni. The accumulated PIXE spectrum (*Q* = 3.5 *μ*C) results in a concentration in weight-ppm of about 2.5 ± 0.6 ppm iron, 1.2 ± 0,6 ppm cobalt and 5.5 ± 0.8 ppm nickel, distributed at random as shown in Fig. 6(c). Note that under the experimental conditions the minimum detection limits for these elements were $\simeq$1 ppm for Fe and Co and $\simeq$2 ppm for Ni.

After subtraction of the diamagnetic part of the *m*(*H*) curve, the obtained FM-like signal at $T\ge 50\,{\rm{K}}$ is shown in Fig. 6(a). According to the results, we obtain a FM contribution at saturation of $m\approx \mathrm{2.5\ }\pm 0.5\,\mu$emu with almost constant temperature dependence up to $T=300\,{\rm{K}}$, indicating that probably the impurity sources are Fe, Ni or Co because of their Curie temperature >300 K. With the values obtained from the average PIXE spectrum and assuming that the contaminants contribute as if they were agglomerates, we obtain a maximum magnetic moment of $m\approx 4\,\mu$emu. This is in reasonable agreement with the FM moment at saturation obtained from the SQUID measurements. Thus, we conclude that FM impurities produce a FM-like signal of the order of a few *μ*emu over the entire temperature range of our experiments. This magnetic moment at saturation is three orders of magnitude smaller than the signals we measured below 25 K for the same sample.

### Optical microscopy, photoluminescence, electron paramagnetic resonance and infrared spectroscopy results

We have used an optical microscope to characterize the surface via cross polarization and the stress distribution within the bulk of the sample. The top and bottom surfaces of the diamond samples present steps of length of a few mm and height of a few micrometers, see inset in Fig. 6(a). These steps do not seem to be related to the crystal axes nor to a polishing procedure. The picture in the inset (upper left) of Fig. 6(a) is an optical microscopy image taken in reflexion from the top surface of the diamond. These steps can easily be seen. The low-right inset in Fig. 6(a) shows an optical microscopy image taken in transmission through crossed polarizers. Such a configuration enables the visualization of the induced birefringence due to the stress within the whole diamond thickness. An inhomogeneous stress distribution can be seen.

A nitrogen defect in diamond can either exists as a single substitutional impurity or in aggregated form. The single substitutional nitrogen has an infrared-active local mode of vibration located at 1344 cm^−1^ ^53^. The electron paramagnetic resonance (EPR) associated to this defect is called P1. The P1 centre is a paramagnetic system with *S* = 1/2 in the ground state^54^. Our results confirm the presence of this centre, see Fig. S17 in the SI for more details. The presence of nitrogen in diamond produces further color centres. To obtain information about the centres present, photoluminescence spectroscopy (PL) at room temperature was used. The PL results indicate the presence of Nitrogen-Vacancy (NV)^−^ centres, see Fig. S17 in the SI for details. Photoluminescence maxima related to other centres are not observable at room temperature. Those measurements done in the ND-S1 sample indicate that it has ≈86 ppm of nitrogen atoms, in agreement with the concentration range given by the company.

Scanning fluorescence confocal microscopy was used to reveal the content of NV centres in the same sample. Indeed, single centres can be easily observed and used as a calibration to retrieve the local NV concentration. We measured a collection of four depth cross-section scans taken at different places of the diamond sample. The fluorescence was measured in the spectral window $500\ldots 800$ nm, which corresponds to NV centres. It was observed that the NV concentration is very inhomogeneous. A region totally free of NV centres was also found, while in other regions a NV density of about 700 NV/*μ*m^3^ (i.e. ~5 ppb) could be found.

**Figure 7:**
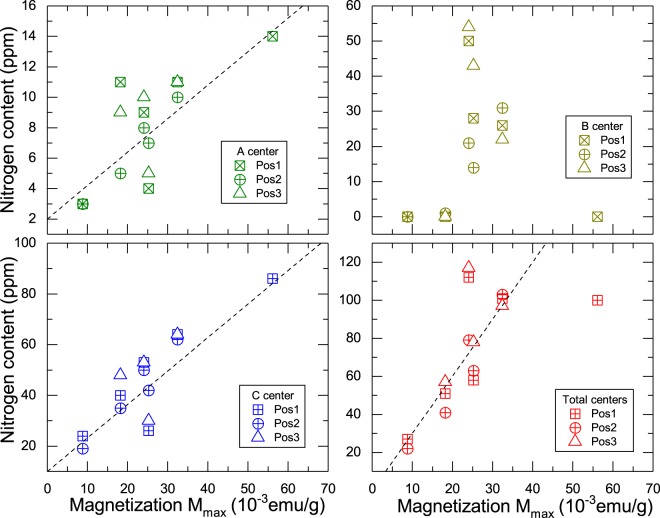
Correlation between the N-content of the three N-centres in diamond (A, B and C) at three different positions in each sample vs. the maximum magnetization value *M*_max_, measured decreasing the field from the maximum applied within the first quadrant at 2 K (see Fig. 2(a)).

We measured the nitrogen-related A-, B- and C-centre concentrations at three different locations in all samples to try to correlate those with some characteristic feature of the observed magnetic field hysteresis. For those measurements we have used infrared spectroscopy, see Section VI in the SI for more details. Figure 7 shows the N content of the three centres and the total amount of them vs. the maximum value of the magnetization *M*_max_, this last obtained from the field hysteresis loop at 2 K (see its definition in Fig. 2(a)). The value *M*_max_ characterizes the whole anomalous magnetic signal. We note that a correlation between *M*_max_ and the C-centres exists and to some extent also with the A-centre, see Fig. 7. Less clear is the correlation of *M*_max_ with the B-centre.

### Magnetization of LSMO/YBCO and Ni/YBCO Bilayers

For comparison and as a support of the reliability of the experimental results obtained for the N-doped diamond crystals, the magnetic properties of bilayers consistent of a ferromagnetic and a superconductor thin film were investigated. The first sample was a 100 nm ${{\rm{YBa}}}_{2}{{\rm{Cu}}}_{3}{{\rm{O}}}_{7-\delta }$ (YBCO) − 50 nm ${{\rm{La}}}_{2/3}{{\rm{Sr}}}_{1/3}{{\rm{MnO}}}_{3}$ (LSMO) bilayer produced *in-situ* by pulsed laser deposition on an STO $\langle 100\rangle$ substrate^55^. In the second bilayer we replaced the LSMO film by a thermally evaporated Ni film of thickness ≈35 nm deposited on a YBCO film of ≈240 nm thickness.

**Figure 8:**
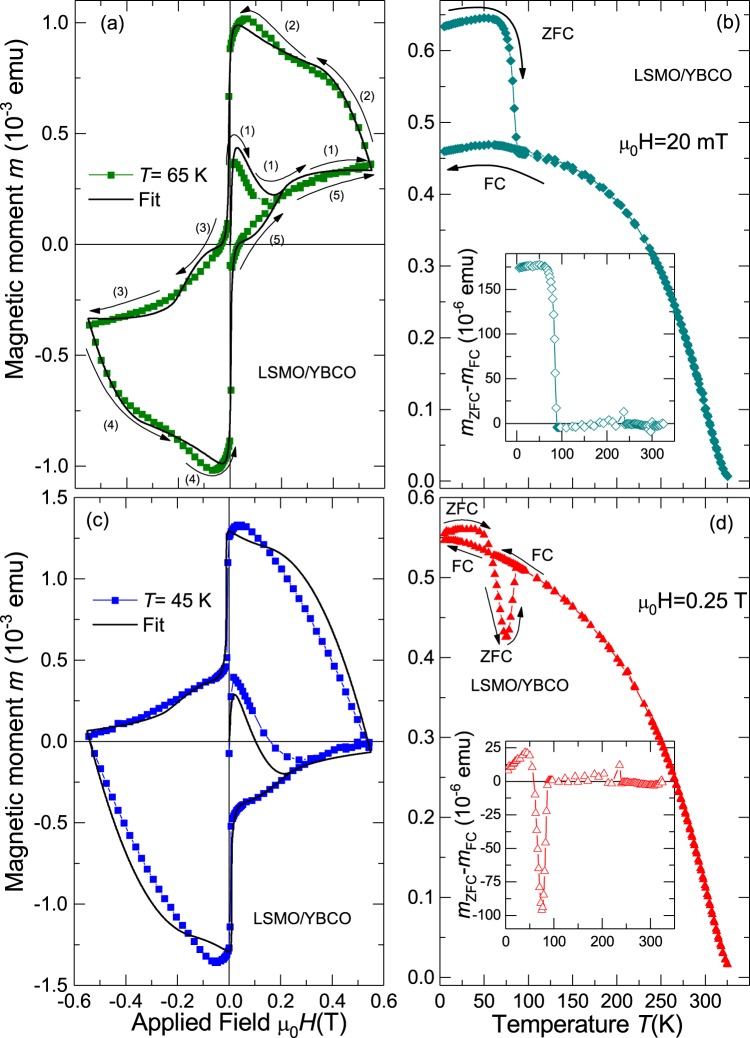
Magnetic moment of the LSMO/YBCO bilayer. The figures (**a**,**c**) show the field hysteresis of the magnetic moment with a similar sequence as used for the diamond samples (see Fig. 1 as example) but with a lower maximum field. The arrows in (**a**) indicates the field sweep direction. The continuous lines were calculated following Eq. (1), see main text for details. Figures (**b**,**d**) show the results of the temperature dependence after ZFC and FC measurements at two different fields. Insets in (**b**,**d**) show the corresponding differences between the ZFC and FC curves. Compare these differences with the ones obtained for N-doped diamond shown in Fig. 3.

The magnetic field was applied always parallel to the main film surface. As example, some results of the magnetic moment as a function of applied field and temperature are presented in Fig. 8 for the LSMO/YBCO bilayer. More results also for the Ni/YBCO bilayer can be seen in the SI.

From the comparison of the hysteresis and temperature dependence of the FM/SC bilayers with the corresponding curves of the N-doped diamond samples we recognize clear similarities, not only in the shape but also in the overall behavior as described for N-doped diamond.

## Discussion

Taking into account the similarities between the magnetization of our N-doped diamond crystals and the FM/SC bilayers and the behavior of the susceptibility shown in Fig. 4 we assume that the anomalous behavior in the diamond samples is due to the presence of both phenomena, SC and SPM. To estimate those contributions we use simple models, which describe the results of the FM/SC bilayer samples very well and extend them to describe the results of the diamond samples changing the FM by the SPM behavior.

We assume that the measured magnetic moment is given by a direct superposition of the following contributions:

1 $$m(H,T)={m}_{dia}(H,T)+{m}_{FM/SPM}(H,T)+{m}_{SC}(H,T),$$

where ${m}_{dia}(H,T)=-\,a(T)H$ is the diamagnetic contribution (*a*(*T*) is a sample dependent parameter added to the contribution of the substrate), ${m}_{FM/SPM}(T)$ the ferromagnetic or superparamagnetic contribution (due Ni, LSMO, or defect-induced regions in the diamond samples) and ${m}_{SC}(T)$ is a superconducting contribution (from the YBCO for the bilayers or doped- or defect-induced in the case of the diamond samples). We model the ${m}_{FM/SPM}(H)$ contribution following Jiles^56,57^ and for the ${m}_{SC}(H)$ a further development within the Bean model by Irie and Yamafuji^58^.

**Figure 9:**
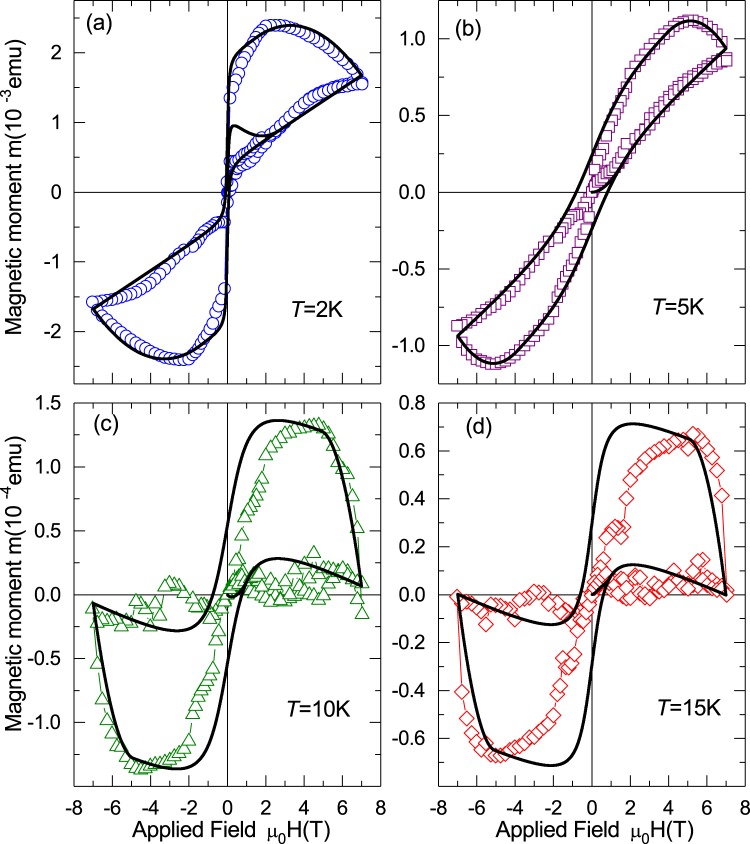
Magnetic moment measured at different temperatures as a function of applied magnetic field of the N-doped diamond sample ND-S1. The lines are calculated from Eq. (1) (see text and the SI for more details).

The model for the ferromagnetic contribution has two main parameters: the saturation magnetization (or magnetic moment) *M*_*FM*_ and the coercive field *B*_*co*_, which we fix to zero for the SPM case. The superconducting contribution (Bean model) is described by the penetration field ${B}_{p}(T)$ and the total hysteresis magnetization width ${M}_{SC}$, defined in the insets of Fig. 9. Within the simple Bean model ${B}_{p}$ as well as the the width of a superconducting field hysteresis ${M}_{SC}$ are parameters proportional to a critical current density, i.e. parameters that depend on the pinning of vortices (or fluxons) and, in general, they decrease with temperature, vanishing at the critical temperature ${T}_{c}$ or at the upper critical field ${B}_{c2}(T)$ of a type II superconductor.

We can determine ${B}_{p}(T)$ as well as ${M}_{SC}$ for the pure YBCO film from its field hysteresis before it was covered with the Ni film (see Fig. S5 in the SI) or by fitting the hysteresis loop of the bilayer leaving those parameter free. The fit results using Eq. (1) are plotted as continuous lines in Figs 8, S11 and S12 for the LSMO/YBCO and in Fig. S13 for the Ni/YBCO bilayers. One can clearly recognize that the simple model assuming two independent contributions to the total magnetic moment describes the basic features of the experimental data. Some of the deviations can be attributed to the time dependence of the magnetization due to flux creep (especially at high enough temperatures), a phenomenon not considered in the fits. The temperature dependence of the penetration field ${B}_{p}(T)$, obtained from the fits of the field hysteresis of the bilayers, is plotted in Fig. S14 of the SI. Additional results on these bilayers are presented in the SI.

The magnetization results of N-doped diamond crystals can be interpreted as due to the simple addition of independent SPM and SC contributions. We tried different combinations and adding different contributions to the diamagnetic background, e.g. two FM contributions, or one SPM and one FM, etc., but none of the combinations can explain the experimental results better than the one following Eq. (1). As in the case of the bilayers, our model describes well the experimental data as shown in Fig. 9.

**Figure 10:**
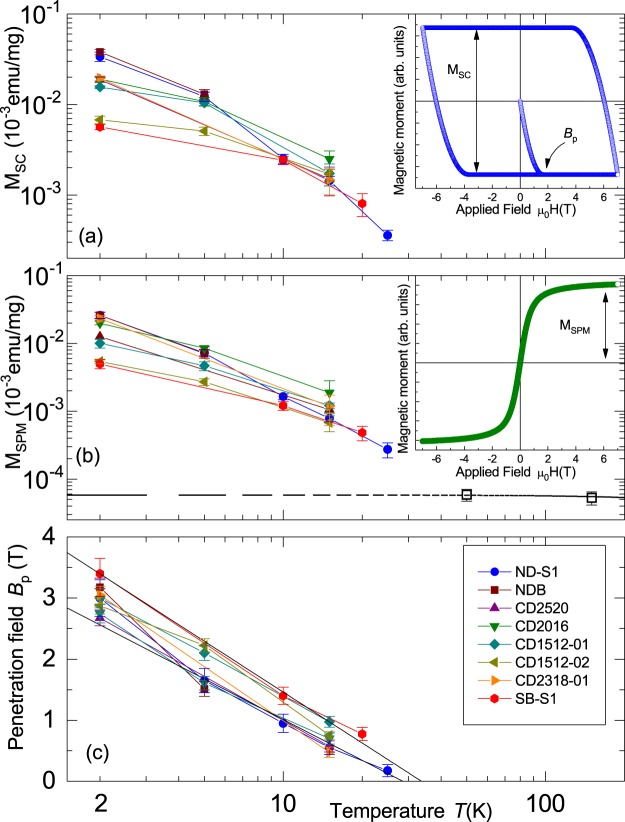
Parameters obtained from the fits to the *m*(*H*) results of all samples investigated in this work. The insets in (**a**,**b**) show the hysteresis loop models for the SC and SPM contributions with the meaning of the plotted parameters. The open squares symbols in (**b**) are the experimental results of the magnetic contribution from the magnetic impurities; the dashed line is calculated assuming that the magnetic contribution follows a Bloch *T*^3/2^ law with the Curie temperature of pure Fe. The lines in (**c**) follow ${B}_{p}(T)={B}_{1}(1+{B}_{2}\,\mathrm{ln}(T/{T}_{c}))$ as fits to the data of the samples ND-S1 and SB-S1 with ${T}_{c}=27\,{\rm{K}}$ and ${T}_{c}=32\,{\rm{K}}$, respectively.

The obtained fit parameters are plotted in Fig. 10. Data were plotted normalized to the sample mass, which evidently is a good scaling factor. According to the fits, the SPM contribution at *T* < 25 K is orders of magnitude larger than the one from the magnetic impurities present in the sample. This is evident considering the experimental values plotted in Fig. 10(b) in comparison with the FM contribution from impurities, which temperature dependence is estimated assuming a Bloch ${T}^{\mathrm{3/2}}$ law.

In contrast to the case of the FM/SC bilayers (consistent of a conventional ferromagnetic and a high temperature superconductor) the penetration field fit parameter ${B}_{p}(T)$ obtained for the N-doped samples satisfies the empirical equation ${B}_{p}(T)\simeq {B}_{1}(1+{B}_{2}\,\mathrm{ln}(T/{T}_{c}))$, with ${B}_{1}$ and ${B}_{2}$ characteristic fields parameters and ${T}_{c}$ a critical temperature above which the SPM and SC contributions become negligible.

From the fit results and the similarities to the behavior of the FM/SC bilayers, we conclude that at temperatures $T\lesssim 25\,{\rm{K}}$, SPM and SC orders appear simultaneously in N-doped diamond with their respective magnitudes proportional to the nitrogen-related centres, see Fig. 7. Note that in the case of the YBCO/LSMO and Ni/YBCO bilayers, the diamagnetic contribution originates from the substrate, in contrast to the case of the N-doped diamond, which is related to the insulating material matrix. This is consistent with the fact that it was not possible to perform DC electrical transport measurements in the N-doped samples due to the high resistivity of the samples. To estimate the relative superconducting mass or volume present in our diamond samples, we take the initial diamagnetic slope at low fields from the SC contribution obtained from the fits of the data at 2 K (see Fig. 10(a)) and compare with the similar signal of the YBCO thin film alone at similar fields and temperatures (Fig. S6 in the SI). We estimate that the superconducting mass in our diamond samples is of the order of 1 *μ*g, i.e. a cube of ~70 *μ*m size, in other words ~10^−4^ to 10^−5^ of the total sample mass. It is clear that this granular superconducting mass cannot be easily detected by electrical resistance measurements.

We have investigated one of the N-doped diamond samples by optical birefringence and the result is shown as inset in Fig. 6. There are clear bright regions indicating that the crystal has internal regions with different optical properties. We speculate therefore that both, the magnetic and superconducting orders could be related to the existence of those defective regions.

Another experimental result supporting our conclusion is the unusual behavior of the magnetic moment at low applied fields, i.e. the ZFC magnetic moment is larger than the one at FC. This can be observed in N-doped diamond (see Fig. 3(a)) and LSMO/YBCO bilayer (see Fig. 8(b)). This behavior can be explained as follows: At low fields, the FM/SPM part is not yet saturated and the SC part is in the Meissner state. This means that when the field is applied the SC part expels the external field, which the FM/SPM part sees as a larger field compared to the applied one and consequently a higher magnetic moment is obtained. During FC, however, the SC part is less diamagnetic such that the local fields generated on the FM/SPM part are smaller than before, thus the FM/SPM contribution diminishes. Our interpretation provides an answer to similar results observed in other carbon-based materials as superconducting phosphorus-doped disordered graphite^59,60^ and graphite^61^.

## Conclusion

The magnetic properties of nitrogen-doped single crystalline diamond samples with N-concentrations below 200 ppm were investigated. All samples show unconventional magnetic moment behavior in the field hysteresis and temperature dependence. The unusual magnetic behavior obtained in the N-doped crystals, including a time dependent magnetic moment, appears below *T* ~ 25 K. Above that temperature all samples behave as a typical undoped diamagnetic diamond. In spite of a large amount of characterization of doped diamond published earlier, the experimental results presented in this work have not been reported yet. Taking into account previous work we propose that the origin of the anomalous magnetization is related to defective localized regions within the dielectric matrix. The main results and open questions of our study are summarized below.

1. The anomalous magnetization signal below 25 K increases with nitrogen concentration, in particular with the nitrogen-related C-centre concentration (Fig. 7). It should be clear that the concentration of nitrogen, which is within a factor of two homogeneously distributed in our diamond samples, appears to be too low to trigger any ordering phenomenon as superconductivity or magnetism, even in granular regions. On the other hand birefringence images clearly indicate the existence of defective, inhomogeneous regions with stress. We suggest therefore that those regions are correlated with the nitrogen concentration and are the origin of the phenomena measured in this study.
2. With a phenomenological model, which considers the measured magnetization as the superposition of a diamagnetic, superparamagnetic (or ferromagnetic) and superconducting contributions (through a Bean-like model), the unconventional results of the field hysteresis loops of all N-doped diamond samples can be well understood.
3. We found that the main characteristics of the measured unconventional behavior in the field and temperature hysteresis of the magnetization resemble the one obtained in ferromagnetic/superconducting oxide bilayers for fields applied parallel to the main area and interface between the two layers. The remarkable similarities in the minutest details of the field hysteresis (Figs 1, 8, S5, S8 and S9) and the temperature dependence of the magnetic susceptibility (Fig. 4) suggest the existence of superparamagnetic and superconducting regions in the N-doped diamond crystals.
4. The obtained phase diagrams for both phases found in the N-doped diamond samples below 25 K (Fig. 10) indicate that the ordering phenomena should have a common origin. In other words, the here found defect-induced superconductivity (DIS) appears to be correlated to defect-induced magnetism (DIM), a nowadays well-known phenomenon in several non-magnetic, including carbon-based, materials. Moreover, the absence of any observable field direction anisotropy as well as the similar values of the saturation magnetization of both contributions (see Fig. 10(a,b)) indicate a field-induced coexistence of both orders. These results would agree with that obtained from STM on the coexistence of superconductivity and magnetic order observed in hydrogen treated boron-doped nanodiamond^31^.
5. The measured time dependence of the magnetization of the N-doped diamond samples indicates the existence of flux creep. This creep follows a stretched time relaxation (Figs 5, S2 and S3). Following several reports of similar flux creep behavior in superconductors with magnetic phases, the observed time dependence is also compatible with the existence of superconducting regions influenced by superparamagnetic domains.
6. That both regions, magnetic and superconducting, are granular-like appears to be a reasonable assumption. On the other hand the used model to fit the field hysteresis, especially the critical Bean-like model to estimate the superconducting contribution, indicates the existence of superconducting shielding currents and field gradients within the grains. This suggests that the size of the superconducting domains should be clearly larger than a few nanometers, as the birefringence image indicates (Fig. 6).
7. Admittedly, to conclude on the existence of superconducting domains only from magnetization measurements is not enough. On the other hand, there are not many other methods one can use to check the existence of granular superconductivity in a dielectric matrix. In fact, it was not possible to measure the resistance of any diamond sample, independently of the positions of the voltage and input current electrodes on them. Future experiments with diamond crystals, also with higher nitrogen concentration, using other scanning techniques are necessary to localize the magnetic and superconducting regions. The experience gained studying the DIM phenomena in nominally non-magnetic samples indicates that key information is obtained not necessarily by local measurements but by macroscopic spectroscopic measurements like XAS and XMCD.

## Methods

We have investigated several nitrogen-doped diamond single crystals as received from the Japanese company SUMITOMO with different macroscopic dimensions and nitrogen content. A picture of one of them is shown as inset in Fig. 6. The synthetic diamond crystals were produced under ultrahigh-pressure and high-temperature (HPHT) conditions and the amount of dispersed nitrogen is between $10\ldots 200$ ppm. One sample (sample SB-S1) was prepared at the Technological Institute for Superhard and Novel Carbon Materials in Moscow. A concentration of substitutional C-nitrogen centres of 86 ppm was measured by electron paramagnetic resonance (EPR) in one of the HPHT crystals, sample ND-S1, discussed in the main text. Magnetic moment measurements of a diamond crystal with nitrogen concentration below 1 ppm did not present any of the interesting features of the nitrogen-doped crystals, within experimental resolution, see those results in Fig. S1 in the SI.

In order to remove any contaminants from the surface, all samples, except sample CD-2016, were cleaned by immersing them in a mixture of 30 mL concentrated sulfuric acid (${{\rm{H}}}_{{\rm{2}}}{{\rm{SO}}}_{{\rm{4}}}$), 10 mL of concentrated nitric acid (${{\rm{HNO}}}_{{\rm{3}}}$) and 10 mL of 60% perchloric acid (${{\rm{HClO}}}_{{\rm{4}}}$). This mixture was heated to ($T\approx 130\,^\circ {\rm{C}}$) for 4 hours under reflux. After cooling to room temperature, the acids were decanted and the diamonds were washed intensively with distilled water. In order to determine the presence of impurities in the investigated samples, particle induced X-ray emission (PIXE) with ppm resolution was used.

The magnetic moment of the samples was measured using a superconducting quantum interference device magnetometer (SQUID) from Quantum Design. Photoluminescence measurements were performed to verify the presence of nitrogen vacancies (NVs) and/or other color centres in our samples. Infrared (IR) spectra were collected with a resolution of 1 cm^−1^ using a Varian FTS6000 Fourier Transform Infrared spectrometer equipped with an UMA600 microscope. From these data the concentration of the A-, B- and C-defects was determined at three different locations of the selected samples, see Section VI in the supplementary information for technical details. The presence of regions with internal stress was investigated by polarized light microscopy.

## Supplementary information

Supplementary Information on:\\ Unconventional Magnetization below 25~K in Nitrogen-doped Diamond provides hints for the existence of Superconductivity and Superparamagnetism
