# Supplementary material for: Unconventional Magnetization below 25 K in Nitrogen-doped Diamond provides hints for the existence of Superconductivity and Superparamagnetism
Source: Sci Rep. 2019 Jun 19;9:8743. doi: 10.1038/s41598-019-45004-6 (PMC6584729; doi:10.1038/s41598-019-45004-6)
Supplement: Supplementary file 1 — Supplementary Information on:\\ Unconventional Magnetization below 25~K in Nitrogen-doped Diamond provides hints for the existence of Superconductivity and Superparamagnetism [file 41598_2019_45004_MOESM1_ESM.pdf]

Supplementary Information on:
Unconventional Magnetization below 25 K in Nitrogen-doped Diamond provides hints for the existence of Superconductivity and Superparamagnetism

J. Barzola-Quiquia,[1,*] M. Stiller,[1] P. Esquinazi,[1,†] A. Molle,[1] R. Wunderlich,[2] S. Pezzagna,[2] J. Meijer,[2] W. Kossack,[3] and S. Buga[4,5]

[1] *Division of Superconductivity and Magnetism, Felix-Bloch-Institute for Solid State Physics, University of Leipzig, 04103 Leipzig, Germany*
[2] *Division of Nuclear Solid State Physics, Felix-Bloch-Institute for Solid-state Physics, University of Leipzig, 04103 Leipzig, Germany*
[3] *Division of Molecular Physics, Peter-Debye-Institute for Soft Matter Physics, University of Leipzig, 04103 Leipzig, Germany*
[4] *Technological Institute for Superhard and Novel Carbon Materials, 7a Centralnaya street, Troitsk, Moscow, 108840 Russia*
[5] *Moscow Institute of Physics and Technology, 9 Institutskiy per., Dolgoprudny, Moscow Region, 141701 Russia*

[*] j.barzola@physik.uni-leipzig.de
[†] esquin@physik.uni-leipzig.de

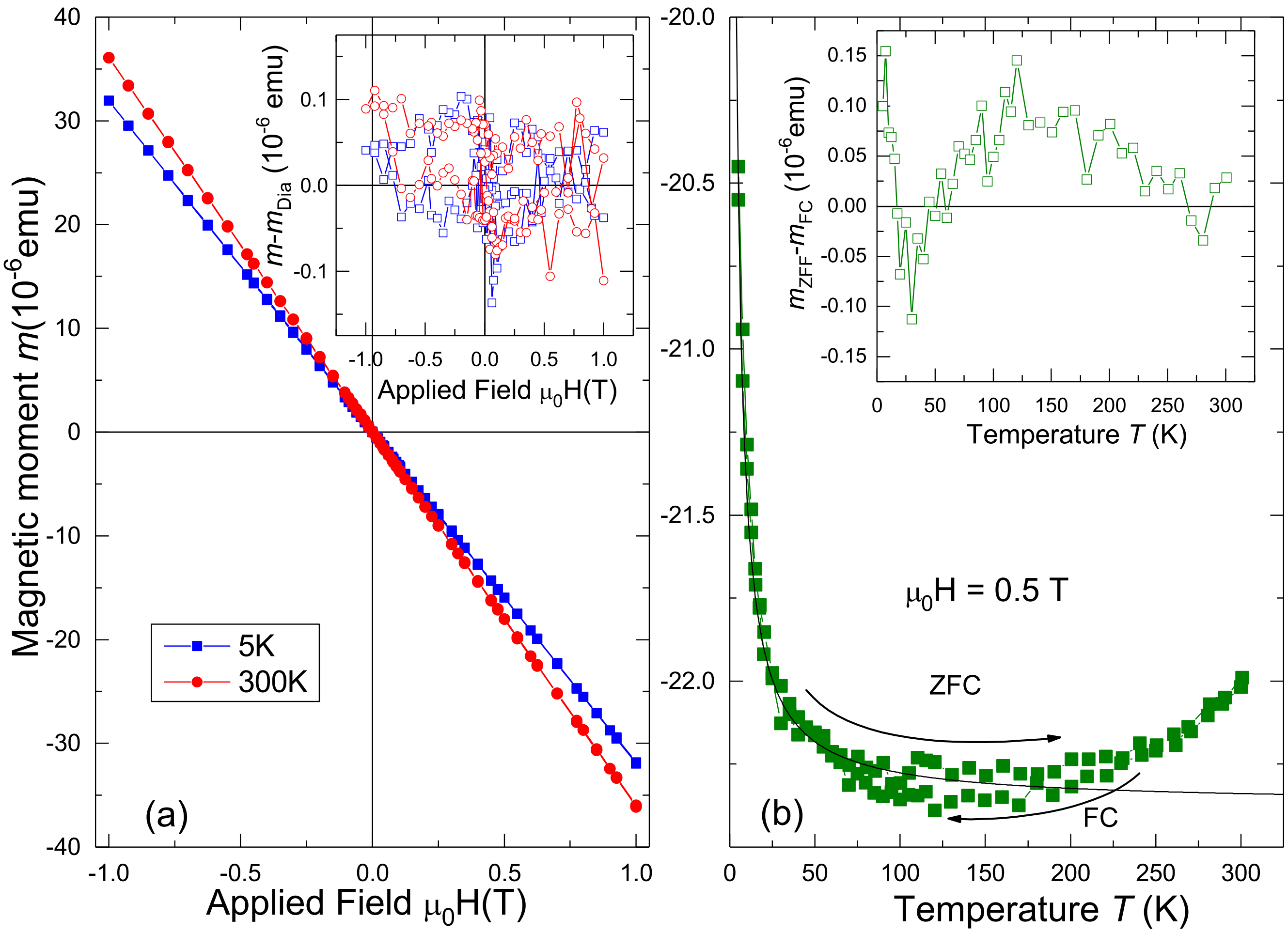

Fig. S 1. Magnetic moment of an undoped diamond sample. (a) Field hysteresis loops at two temperatures. The inset shows the same results but after subtraction of the diamagnetic linear in field contribution. (b) The temperature loop, i.e. ZFC and FC curves. The continuous line is a fit of the data points below 100 K to the Curie-law. The inset shows the difference between the ZFC and the FC results.

I. MAGNETIZATION OF AN UNDOPED DIAMOND SAMPLE

In the main text we have mentioned that the magnetization of pure, undoped diamond behaves as a diamagnetic material in the temperature range of 2 K to 300 K. The results of the field hysteresis at two temperatures and also as a function of temperature are shown in Fig. S1. These results indicate that after subtraction of the diamagnetic contribution the small hysteresis loop (see inset Fig. S1(a)) is of the order of 10^{-7} emu, i.e. about three orders of magnitude smaller than the hysteresis found in N-doped samples below ~ 20 K. The resolution of the used SQUID device is $\sim 2 \times 10^{-8}$ emu [1]. The magnetic moment shows a weak temperature dependence above 50 K. At lower temperatures, it shows a weak paramagnetic contribution probably due to natural defects present in the sample; the estimated concentration of paramagnetic centres obtained from this dependence is extremely small (< 0.01 ppm). The small differences of the order of 10^{-7} emu between the zero field cooled (ZFC) and field cooled (FC) curves (see inset in Fig. S1(b)) are orders of magnitude smaller than in N-doped samples. From all these results we conclude that pure diamond without nitrogen doping shows basically a diamagnetic behavior in the whole temperature range, without any signs of magnetic or superconducting order within experimental resolution.

II. TIME DEPENDENT MAGNETIZATION

The magnetization as a function of time was measured in the N-doped diamond sample ND-S1 at different temperatures. The corresponding results are plotted in Fig. S2. A clear time dependence below $T \simeq 25$ K is observed.

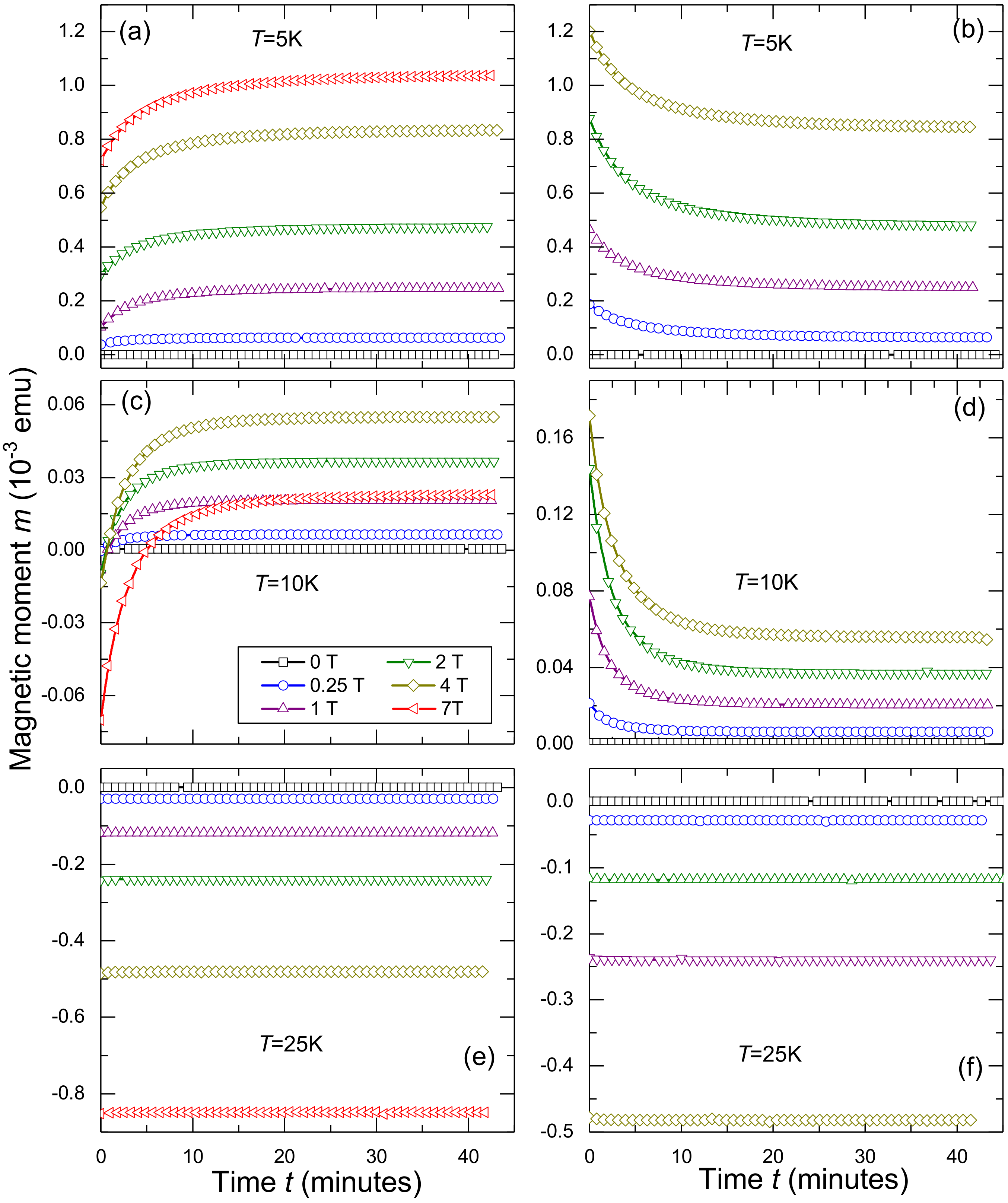

Fig. S 2. Magnetic moment as a function of the time at different constant temperatures and magnetic fields in sample ND-S1. The results (a), (c) and (e) were measured after the magnetic field was applied. The results in (b), (d) and (f) were measured after the magnetic field was reduced from the previous field, e.g., the data at 4 T were obtained immediately after the field was reduced from 7 T.

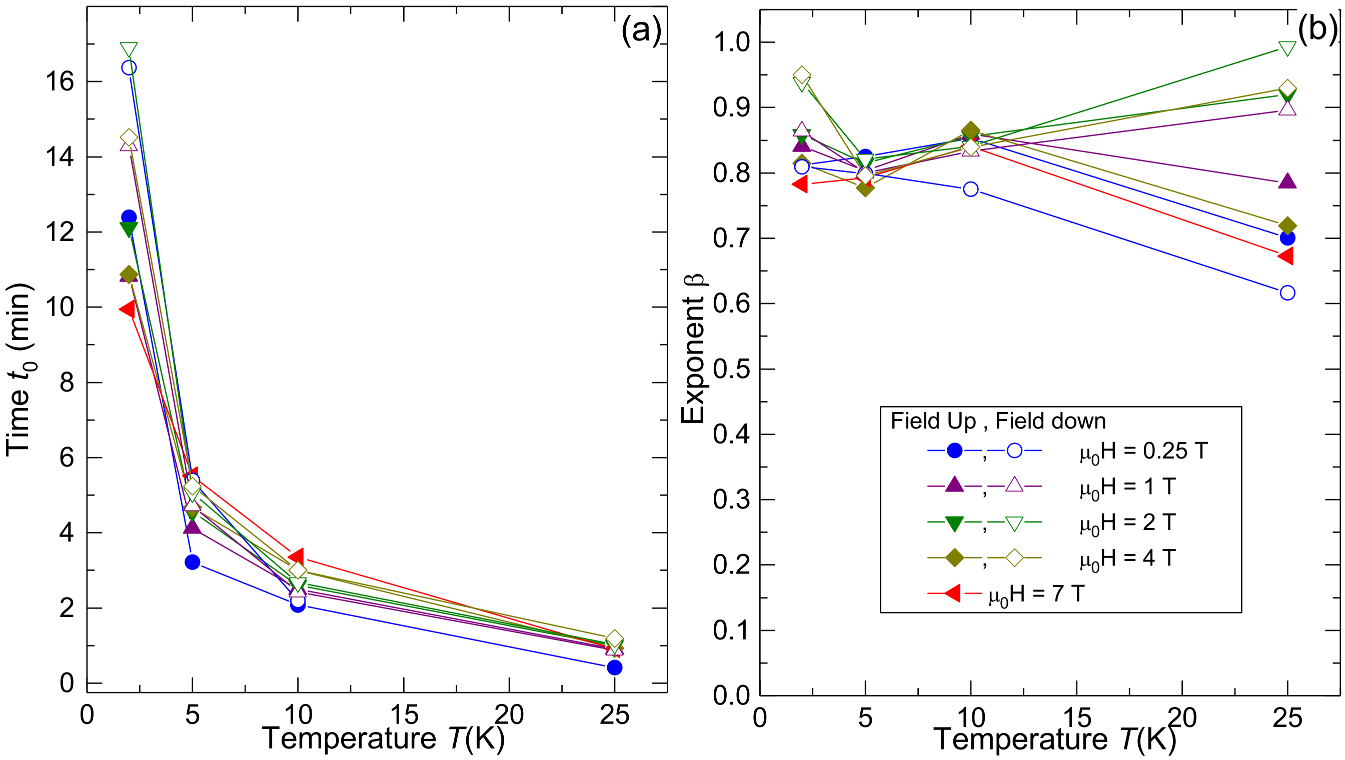

Fig. S 3. Parameters obtained from the fits of the time dependence of the magnetic moment $m(t)$ of sample ND-S1 to a stretched exponential function as explained in the main text, at different temperatures and applied magnetic fields. In (a), the characteristic time t_0 and in (b), the parameter β are shown. The size of the symbols approximately corresponds to the fitting error.

Above 25 K, the N-doped sample behaves as in the case of undoped diamond, without any time dependence within experimental error. Fig. S 3 shows the parameters t_0 and β as a function of temperature obtained from the fits to the stretched exponential function of sample ND-S1 (see Fig. 5 in the main text).

The time dependence of the magnetization below $\sim$ 25 K influences to some extent the shape of the hysteresis loop, as the results obtained at different waiting times shown in Fig. S4 indicate. This time relaxation is not taken into account in the fits of the field hysteresis. At this stage it is better to minimize the number of free parameters to convince the reader on the main contributions that influence the field hysteresis loops.

III. TEMPERATURE AND FIELD DEPENDENCE OF THE MAGNETIC MOMENT OF THE DIAMOND SAMPLES WITH DIFFERENT NITROGEN CONTENT

In the present work a total of 8-samples with different N-content were investigated, seven of them were obtained by the Japanese company Sumitomo and one was delivered from the Russian Institute. The results of the six samples not shown in the main text can be seen in Fig. S5. The samples in (a) to (e) are labeled according to their original identification number from the Sumitomo company. As the company indicates, the N-content in the diamond samples is between 10 ppm to 100 ppm. The results presented in Fig. S5 are the raw data, very similar to the one presented in the main text. The differences are related to the N-content and not to the different masses of the samples, i.e. the samples with high N-content show a larger absolute hysteresis (e.g. samples CD2016 and CD2318-01) than the ones with less N-content (e.g. sample CD1512-02). Figure 7 of the main text shows the correlation of the magnetization at 2 K with the N-content of all samples.

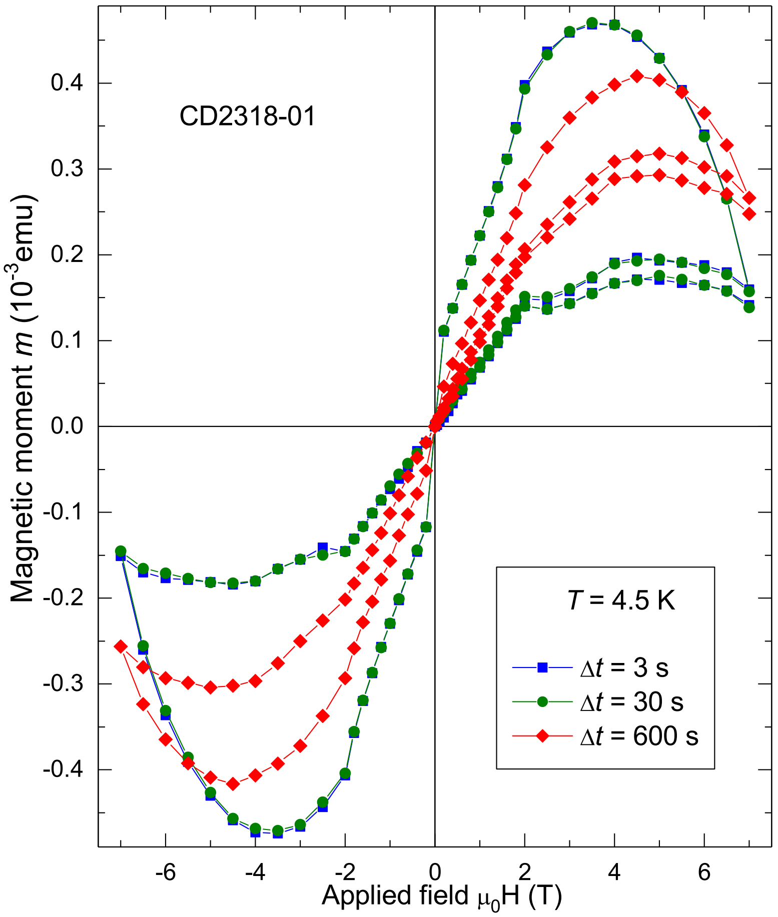

Fig. S 4. Field hysteresis loops measured at different waiting times between points in sample CD2318-01 at 4.5 K. The time relaxation effects are clearly observed after several minutes waiting time between the points.

IV. MAGNETIC MOMENT OF THE YBCO FILM AND THE BILAYERS LSMO/YBCO AND Ni/YBCO

To support our interpretation regarding the coexistence of superconductivity and superparamagnetism in the N-doped diamond samples, we have measured bilayers with a superconductor and a ferromagnetic material. Two different ferromagnetic materials and the high temperature superconductor $YBa_2Cu_3O_{7-x}$ (YBCO) were used. One investigated bilayer had ferromagnetic $La_{2/3}Sr_{1/3}MnO_3$ (LSMO) and the second sample nickel (Ni). In the first sample both materials were deposited by pulsed laser deposition (PLD) [2]. In the second sample YBCO was deposited first by PLD and the Ni film afterwards by thermal evaporation. Because of the growth conditions, the sample LSMO/YBCO was made in one process, but in the case of the sample Ni/YBCO, we had the chance to measure initially the YBCO sample before depositing the Ni. The results of the YBCO film alone are shown in Fig. S 6, after subtraction of the diamagnetic contribution of the substrate.

The results shown in Fig. S 6 reveal the typical behavior of the magnetic moment m(H) of YBCO, i.e. at low fields we observe a diamagnetic linear response with a clearly change of slope at fields near a penetration field B_p. The obtained results shown in Fig. S14 indicate that the penetration field follows the empirical equation $B_p(T) = B_0[1 - (T/T_c)^2]$ with $B_0 \simeq 0.2$ T and a critical temperature of $T_c = 90$ K, in good agreement with literature and the T_c obtained from $M(T)$ shown in Fig. S 7 . The magnetic moment of the YBCO sample as a function of temperature at two different applied fields is shown in Fig. S 7. From the $m(T)$ we estimate a critical temperature of $T_c = 89 \pm 0.5$ K and $T_c = 84 \pm 0.5$ K at 5 mT and 0.25 T.

After this characterization of the YBCO film alone, we have measured the FM/SC bilayers. The field hysteresis loops of the bilayer LSMO/YBCO are plotted in Fig. S8 and of the Ni/YBCO bilayer in Fig. S9.

The results presented in Fig. S 8 and Fig. S 9 are the raw data, i.e the results contain the diamagnetic contribution from the substrate. We have also measured the magnetic moment as a function of temperature $m(T)$ (ZFC and FC)

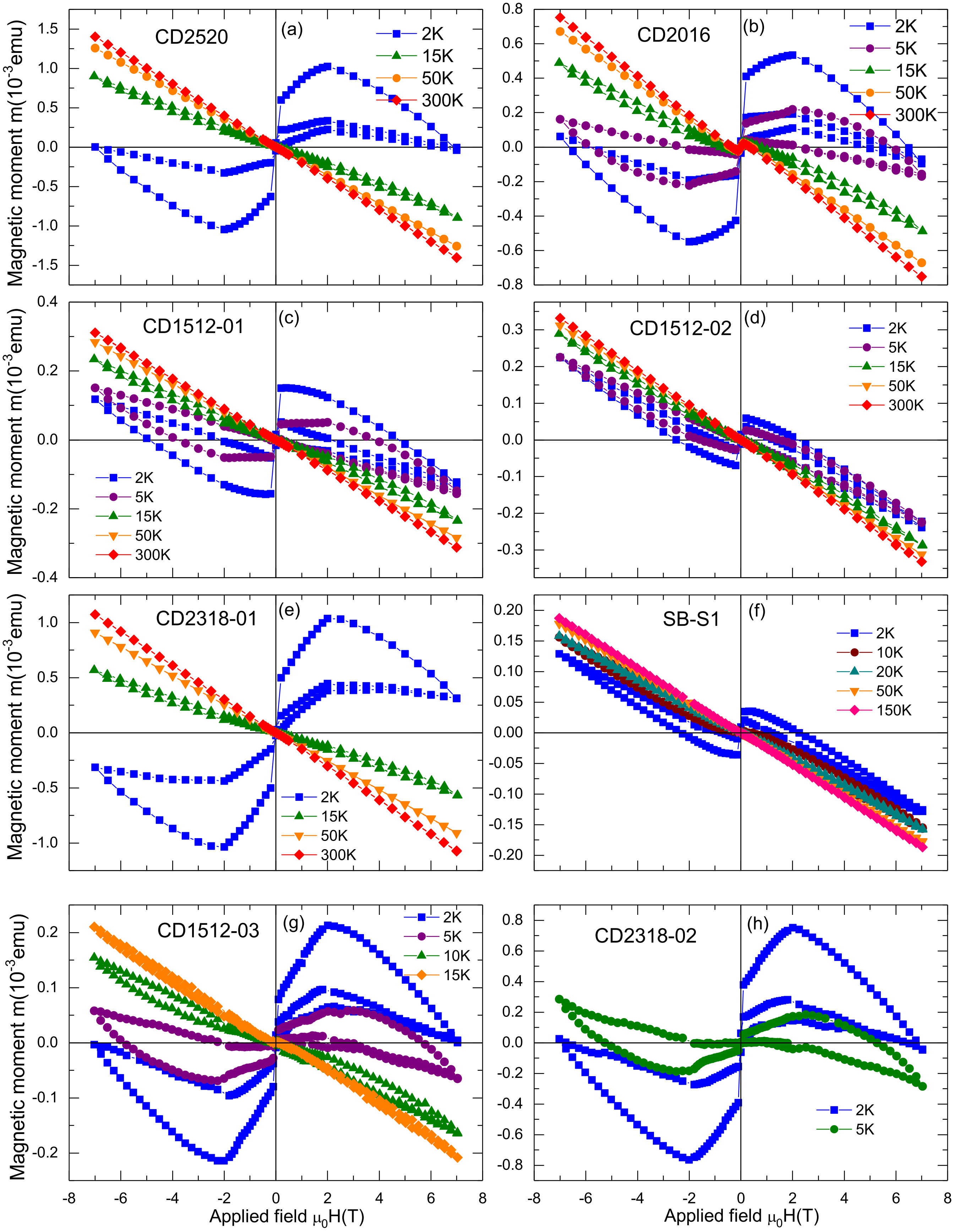

Fig. S 5. Raw data of the magnetic moment $m(H)$ of all N-doped samples investigated in this work. The mass of the samples is CD2520: 44.1 mg, CD2016: 20.1 mg, CD1512-01: 9.58 mg, CD2318-01: 34.05 mg, CD1512-03: 9.99 mg, CD2318-02: 34.62 mg, CD1512-02: 9.89 mg, SB-S1: 6.2 mg .

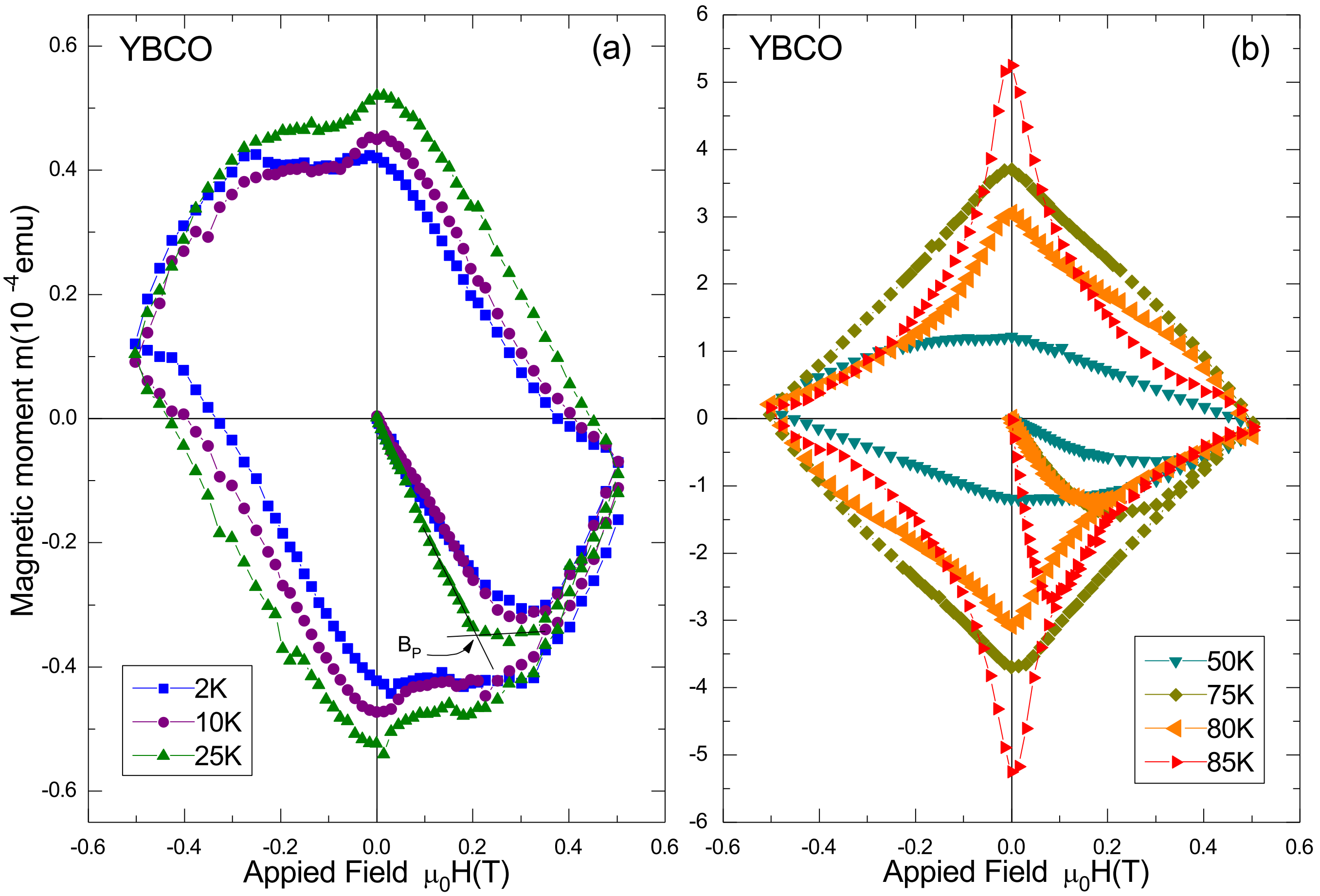

Fig. S 6. Magnetic moment as a function of the magnetic field applied parallel to the main area of the YBCO film measured before the Ni film was deposited.

and the results are shown in Fig. S 10, which provide a superconducting transition temperature of $T_c = 86 \pm 0.5$ K and $T_c = 90 \pm 0.2$ K for the LSMO/YBCO and Ni/YBCO bilayers, respectively.

The temperature dependence $m(T)$ reveal an unusual behavior during the ZFC and FC measurement, even with crossing, see Fig Fig. S 10(b). From $m(T)$ and especially the field hysteresis $m(H)$ of both bilayer samples we clearly recognize a very similar behavior to that of the N-doped diamond samples, see Figs. 3 and 9 in the main text and several figures in this supplementary information.

V. MODELING THE MAGNETIC MOMENT FIELD HYSTERESIS LOOPS

In order to describe the magnetic moment of the LSMO/YBCO and Ni/YBCO bilayers and of the N-doped diamond samples we have used a simple model. The total magnetic moment is as a superposition of three components, first the diamagnetic contribution, coming from the substrate, in the case of the bilayers, or from the diamagnetic part of the diamond matrix $m_{dia}(H)$. Second, a ferromagnetic or superparamagnetic contribution $m_{FM/SPM}(H)$ and the third a superconducting contribution $m_{SC}(H)$, as follow:

$$m(H) = m_{dia}(H) + m_{FM/SPM}(H) + m_{SC}(H)\,, \tag{1}$$

whereas for the diamagnetic term we used a linear in field $m_{dia}(H) = -a(T)$H, where $a(T)$ is a sample and temperature dependent diamagnetic slope. For the FM/SPM contribution we used the phenomenological model proposed by Jiles et al. [3, 4] for the case of an isotropic material in which the magnetization has essentially no preferred direction. The

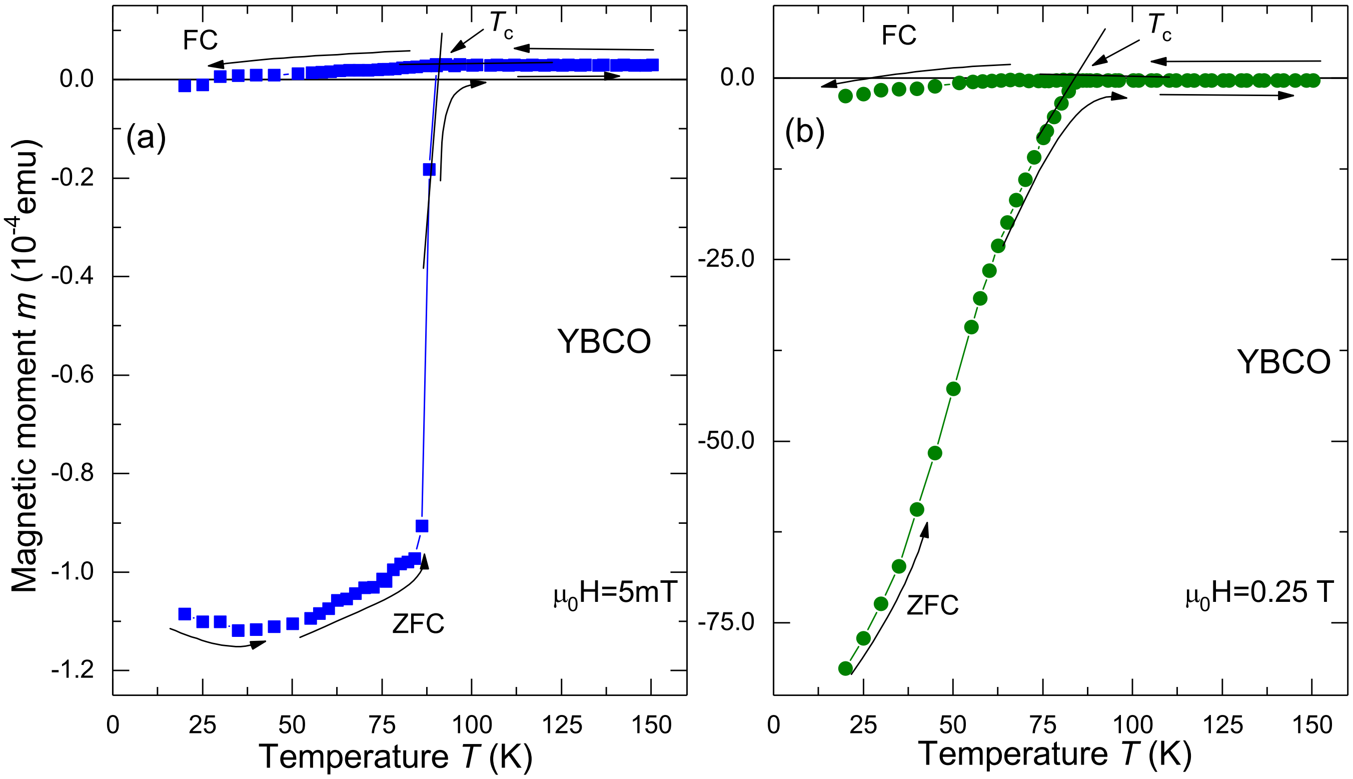

Fig. S 7. Magnetic moment of the YBCO sample as a function of temperature at two applied fields. The bent arrows in (a) and (b) indicate the direction of the temperature sweep at the ZFC and FC measurements.

equation for the FM/SPM part is given as follow:

$$m_{FM/SPM}(H) = m_0 \left[\coth\left(\frac{H + \alpha M}{a} \right) - \frac{a}{H + \alpha M} \right], \tag{2}$$

with m_0 the saturation magnetic moment, the fit parameters α and a (as well as dM/dH) are described in [4]. The main differences in the simulation of the FM and SPM contributions is that in the last no field hysteresis is assumed and the low field susceptibility depends strongly on temperature.

For the SC contribution we have used the theory developed by Irie and Yamafuji [5], which is a further development of the Bean model, and the equation is as follow:

$$m_{SC}(H) = H_p \frac{2-\gamma}{3-\gamma} \left[\left(\frac{H}{H_p} \right)^{3-\gamma} - c(H) \right] - |\frac{H}{H_p}|, \tag{3}$$

with $H_p = B_p/\mu_0$ the penetration field, and γ a free pinning dependent parameter ($\gamma = 1$ corresponds to the original Bean model) and $c(H)$ a complex function, see [5]. Using the above equations for each contribution in Eq. (1) we have modeled the experimental results of the bilayers and of the N-doped diamond samples (assuming for simplicity $\gamma = 1$ in this last case).

A. Results of the FM/SC bilayers

The first results we present in Fig. S11 are of the LSMO/YBCO sample at $T = 65$ K, where the continuous line is the result of the fit obtained from the addition of the three contributions shown in (b) to (d). Fig. S12 shows the field hysteresis loops at four different temperatures and the corresponding fits for the same bilayer. Fig. S13 shows the results of the Ni/YBCO bilayer. The fitting curves describe the experimental results rather well and at all temperatures.

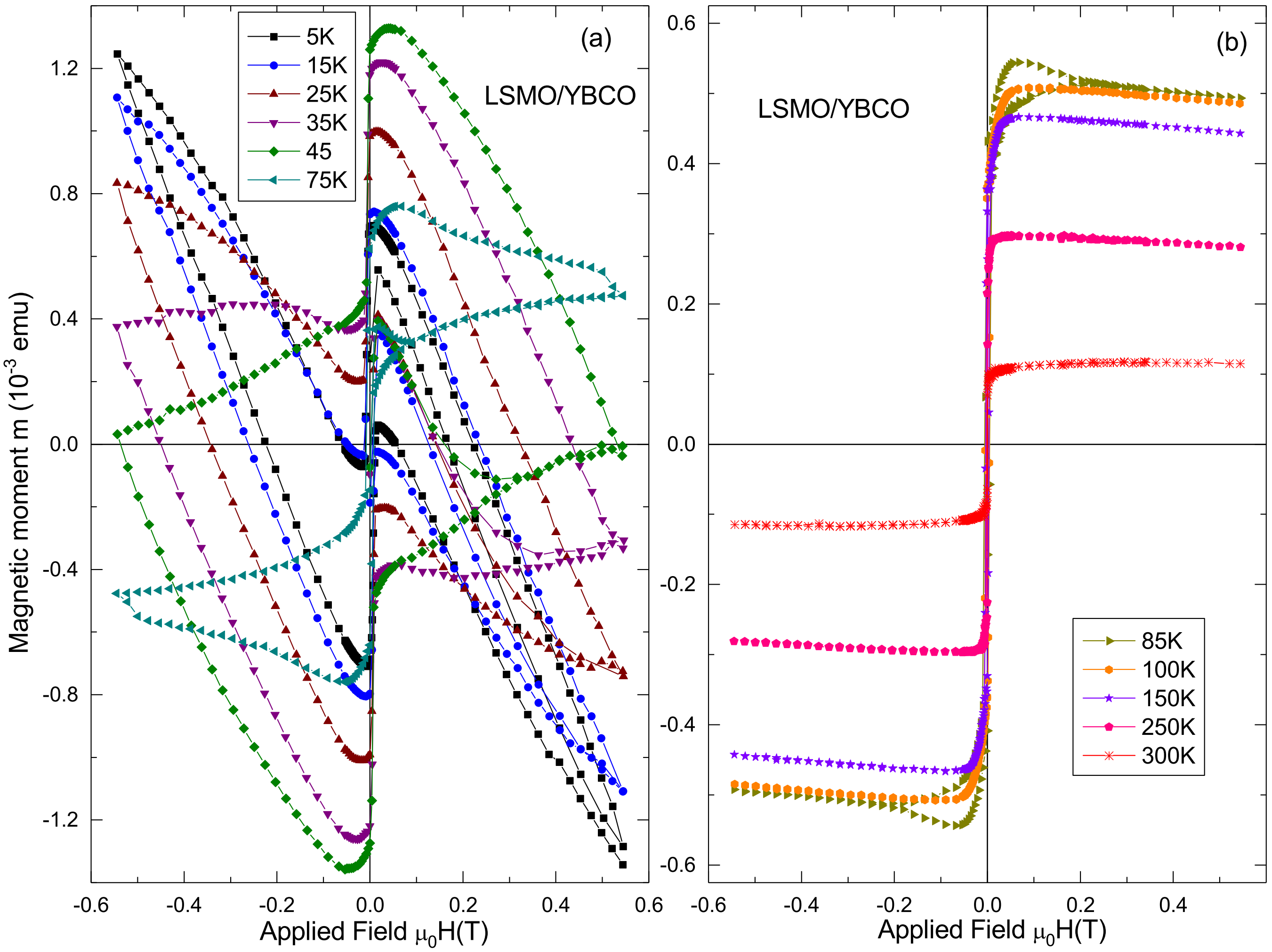

Fig. S 8. Field loops of the magnetic moment of the bilayer LSMO/YBCO at different constant temperatures; in (a) below the superconducting critical temperature T_c and in (b) around and above T_c.

For both bilayers we observe that the obtained penetration field $B_p = \mu_0 H_p$ from the fits, see Fig. S14, follows the empirical equation $B_p(T) = B_0(1-(T/T_c)^2)$ with critical temperatures similar to that obtained from the magnetization measurements of the YBCO layer alone, see Fig. S7. The penetration field $B_p(T)$ from the fits is of the same order as the one we obtain from the single YBCO layer. We note that the values of $B_p(T)$ obtained from the YBCO alone and shown in Fig. S14 were multiplied by a constant factor $\simeq 1.5$. This change in the values of B_p can be partially attributed to some influence of the FM layer on the vortex pinning in the YBCO layer.

B. Results of the N-doped diamond samples

In Fig. S 15 we have plotted all the field hysteresis and the fit results obtained at $T = 2$ K of the investigated N-doped diamond samples. The fit results describe well the experimental results, supporting our interpretation that the N-doped diamond samples contain regions with SPM and SC properties. Some of the differences between the experimental results and the results of the model can be due to the time dependence due to creep, which is not consider in the calculations.

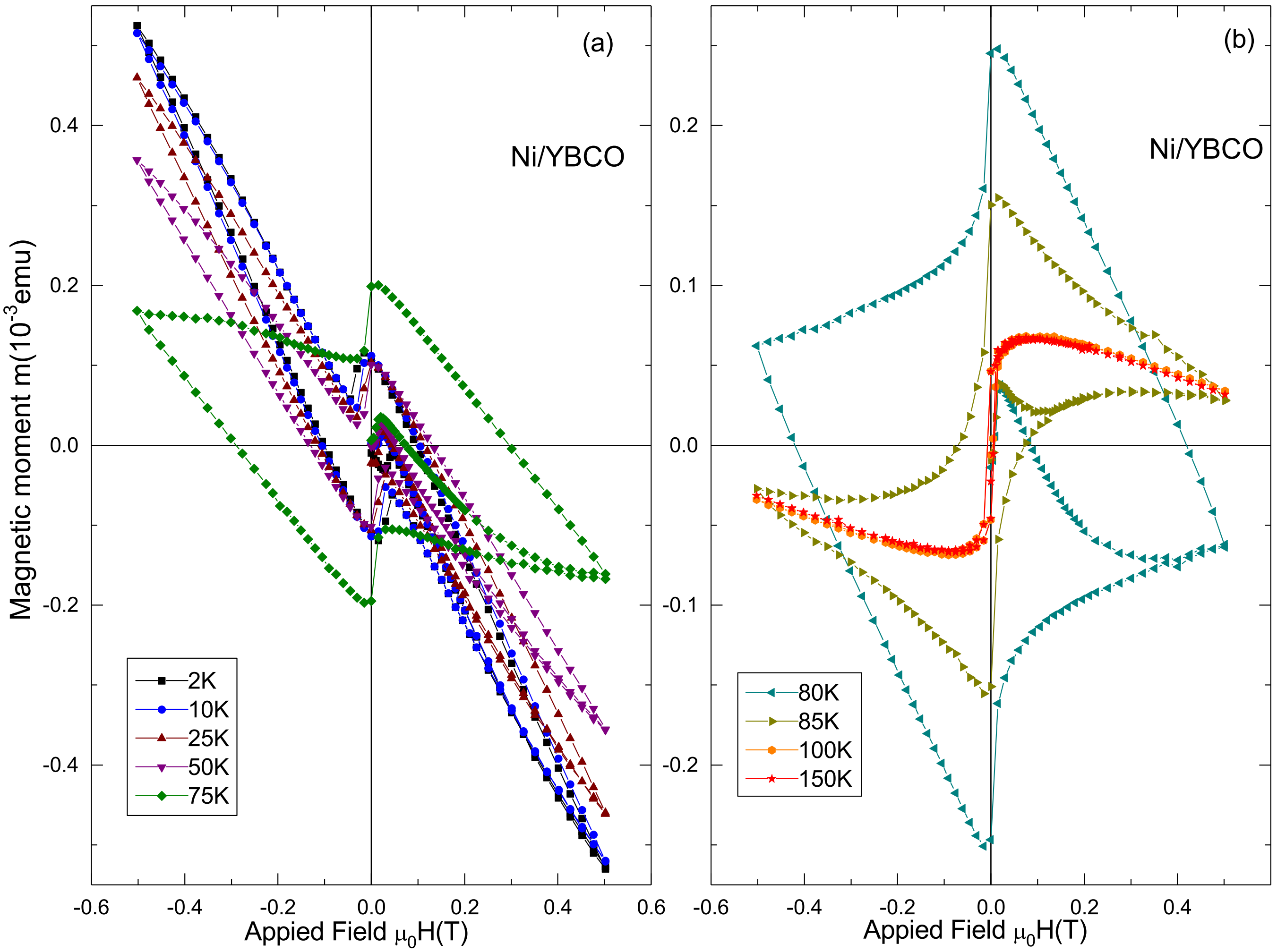

Fig. S 9. Same as in Fig. S8 but for the Ni/YBCO bilayer. In (a) we show the results below T_c and in (b) around and above T_c.

VI. INFRARED SPECTROSCOPY

Infrared (IR) spectra are collected with a resolution of 1 cm^{-1} using a Varian FTS6000 Fourier Transform Infrared spectrometer equipped with an UMA600 microscope. Data were corrected with a linear baseline between 980 cm^{-1} and 1400 cm^{-1} (Fig. S 16 (e1)). The concentration of the A-defects (c_A) was determined according to the absorption coefficient in cm^{-1} at 1282 cm^{-1} : $\mathrm{c_A} = [16.5 \pm 2]\mathrm{ppmcm}\alpha_{1282}\,\mathrm{ppm}$ [6, 7]. The concentration of the C- (P_{1-}) defects in ND-S1 sample as determined from EPR (86 at ppm) is used to calculate the molar absorption coefficient, $\epsilon_\mathrm{C} = 44.6$ ppm cm, of the C-defects[8] at 1344.5 cm^{-1}: $c_\mathrm{C} = \epsilon_\mathrm{C}\alpha_{1344.5}$.

To the best of our knowledge, no molar absorption coefficients for B-type defects are reported for type IaAB diamonds [9], and apparently no one has yet managed to measure a real concentration of nitrogen in diamond [7]. We therefore approximate the concentration of B-defects, c_B, by the absorption of the B-specific peak (ν_B) at 1332.3 cm^{-1}. In this spectral region also A defects add some absorbance [6], which needs to be subtracted before. For all samples, especially those without ν_B (Fig. S 16 (e1)), a step in absorption coefficient, α_step, exists between 1400 cm^{-1} and 1316 cm^{-1}, and its height is proportional to c_A:

$$\begin{aligned}\alpha_\mathrm{step} &= \alpha_{1316}\ \mathrm{cm}^{-1} - \alpha_{1400}\ \mathrm{cm}^{-1} \\ &= [0.13 \pm 0.03]\ \mathrm{ppm}^{-1} c_\mathrm{A}\,.\end{aligned}$$

Consequently, we assign α_step, to the A-defects, and subtract it before determining c_B (Fig. S 16 (e2)).

The molar absorption coefficient of the B-defects, ϵ_B at 1332.3 cm^{-1}, is estimated from the total amount of dispersed

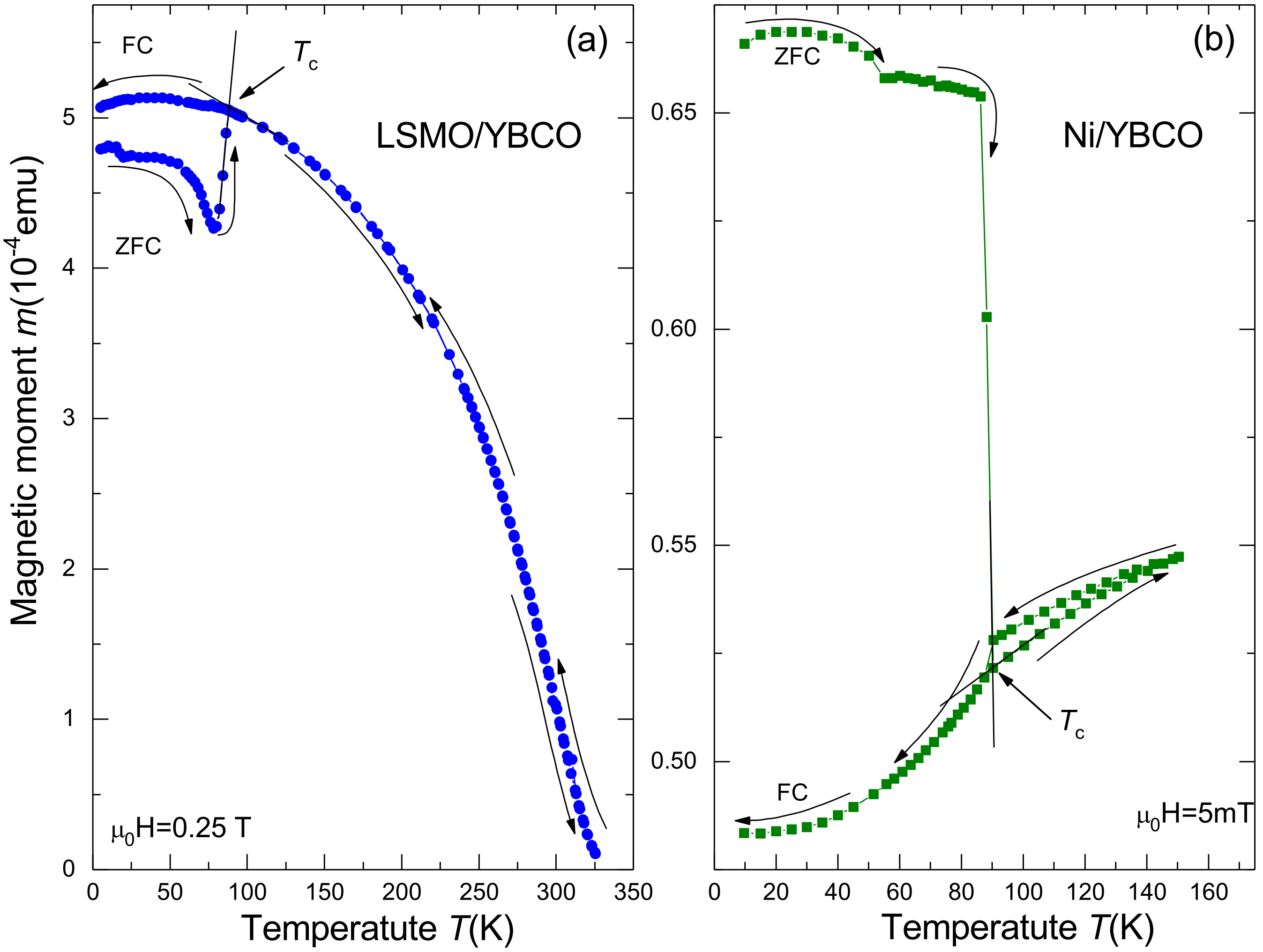

Fig. S 10. Magnetic moment as a function of the temperature (ZFC and FC) of the (a) LSMO/YBCO and (b) of the Ni/YBCO bilayer.

nitrogen $c_{\rm tot} = c_{\rm A} + c_{\rm B} + c_{\rm C}$. For ND-S1 no B-defects are found, and hence $c_{\rm tot} = c_{\rm A} + c_{\rm C} = 100$ ppm. This value is in agreement with the manufacturers information. Assuming the same $c_{\rm tot}$ for sample 2318-01 yields:

$$\begin{aligned} c_{\rm B} &= c_{\rm tot} - c_{\rm A} - c_{\rm C} \\ &= \epsilon_{\rm B}(\alpha_{1332} - \alpha_{\rm step}) \\ \epsilon_{\rm B} &= 71.7 \text{ ppm cm}\,, \end{aligned}$$

where $\epsilon_{\rm B}$ is strongly dependent on $c_{\rm tot}$. For example, for $c_{\rm tot} = 150$ ppm one finds $\epsilon_{\rm B} = 210$ ppm cm. But in this case $c_{\rm tot}$ exceeds 200 ppm for sample 1512-03, a concentration twice as high as given by the manufacturer.

In summary, we can determine the concentration of A- and C-defects with an accuracy about ±10% based on published studies. We propose a new approach to determine the concentration of B-type defects in type IaAB diamonds, which suffers from the uncertainty of the total nitrogen concentration. But the approach allows for a quantitative comparison of $c_{\rm B}$ between different samples. Furthermore, no indications of boron [9] or nitrogen-platelets [8] (area defect) were found in the IR-spectra.

VII. ELECTRON PARAMAGNETIC RESONANCE AND PHOTOLUMINESCENCE

Electron paramagnetic resonance results shown in the inset of Fig. S17 confirm the existence of the well-known P1 centre in our samples. Photoluminescence spectroscopy measurements were done with two different wavelengths,

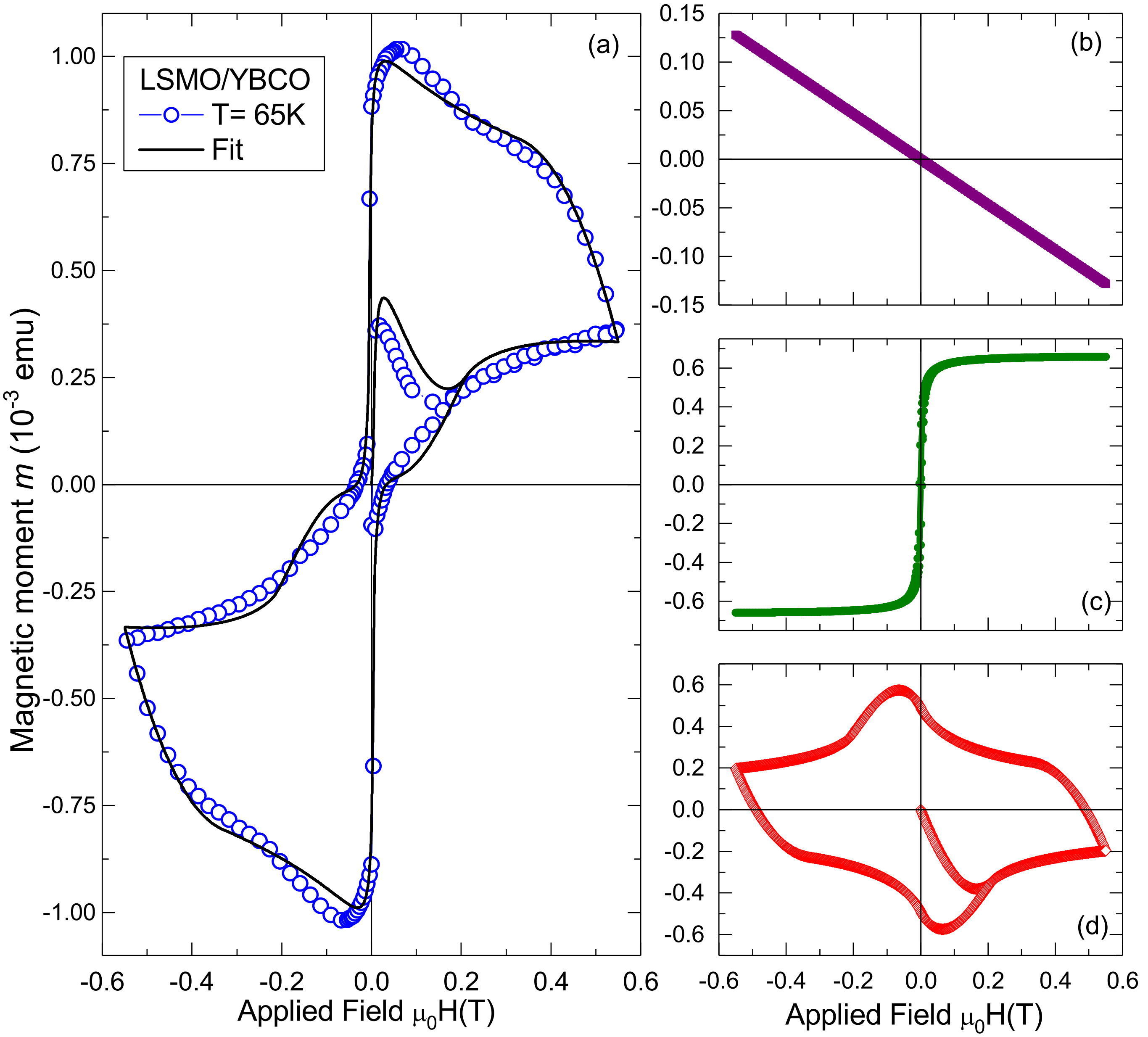

Fig. S 11. Field hysteresis loop of the LSMO/YBCO bilayer at 65 K. In (a) open symbols are the experimental results and the line is the result of the modeling explained in the text. From (b) to (d) we show the different contributions used to model the experimental results: (b) $m_{dia}(H)$, (c) $m_{FM}(H)$ and (d) $m_{SC}(H)$.

$\lambda_1 = 488$ nm and $\lambda_2 = 532$ nm. The results are plotted in Fig. S 17, the first and second Raman order as well as the typical N-centres are indicated with vertical lines.

[1] J. Barzola-Quiquia, P. Esquinazi, M. Rothermel, D. Spemann, A. Setzer, and T. Butz, Nucl. Instrum. Methods Phys. Res. B **256**, 412 (2007).

[2] S. Soltan, J. Albrecht, and H.-U. Habermeier, Phys. Rev. B **70**, 2144517 (2004).

[3] D. C. Jiles and J. B. Thoelke, IEEE Transactions on Magnetics **25**, 3928 (1989).

[4] D. C. Jiles, J. B. Thoelke, and M. K. Devine, IEEE Transactions on Magnetics **28**, 27 (1992).

[5] F. Irie and K. Yamafuji, Journal Phys. Soc. Japan **23**, 255 (1976).

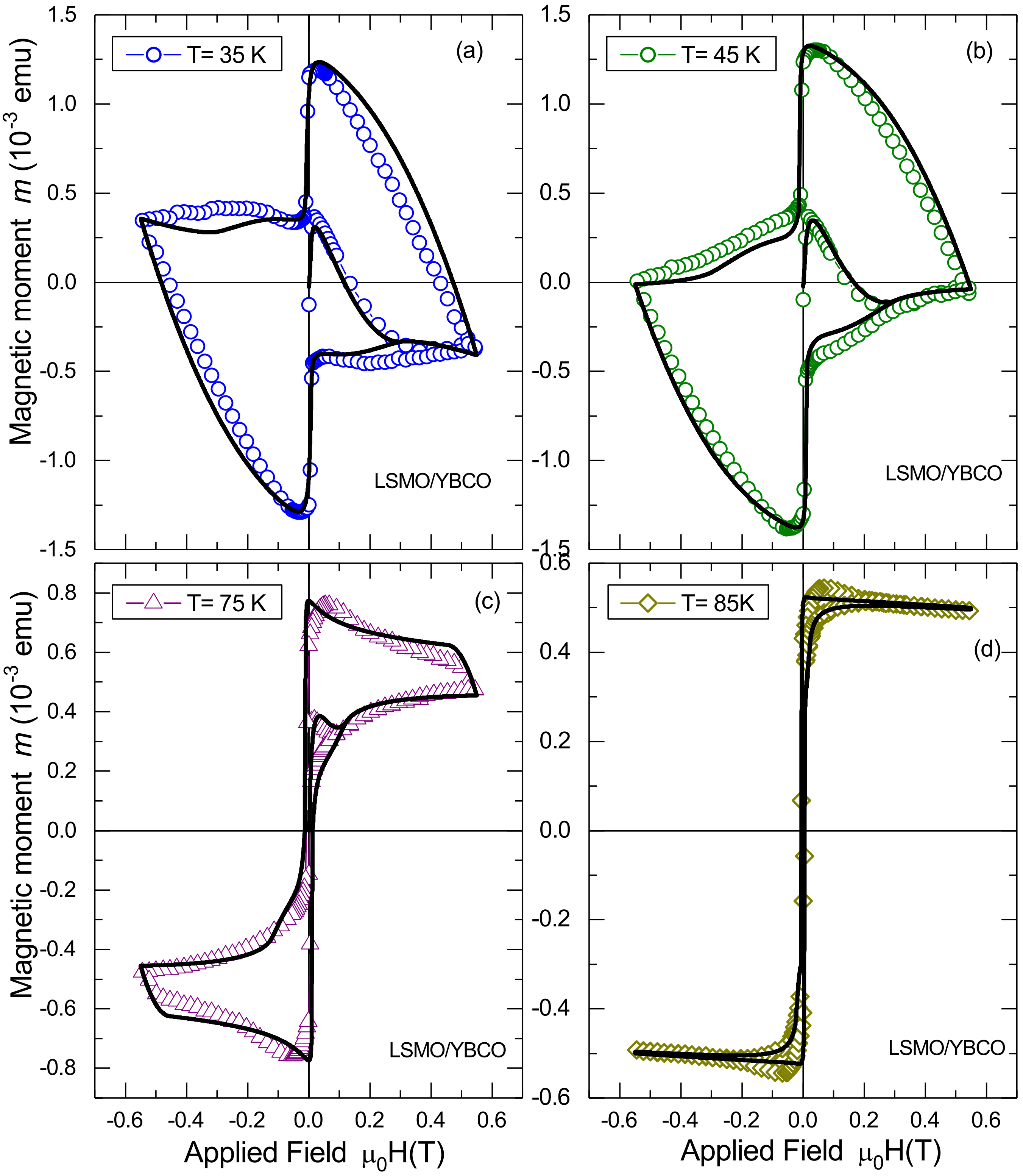

Fig. S 12. Field hysteresis loops of the LSMO/YBCO bilayer at different temperatures with the results of the model using Eqs.(1-3).

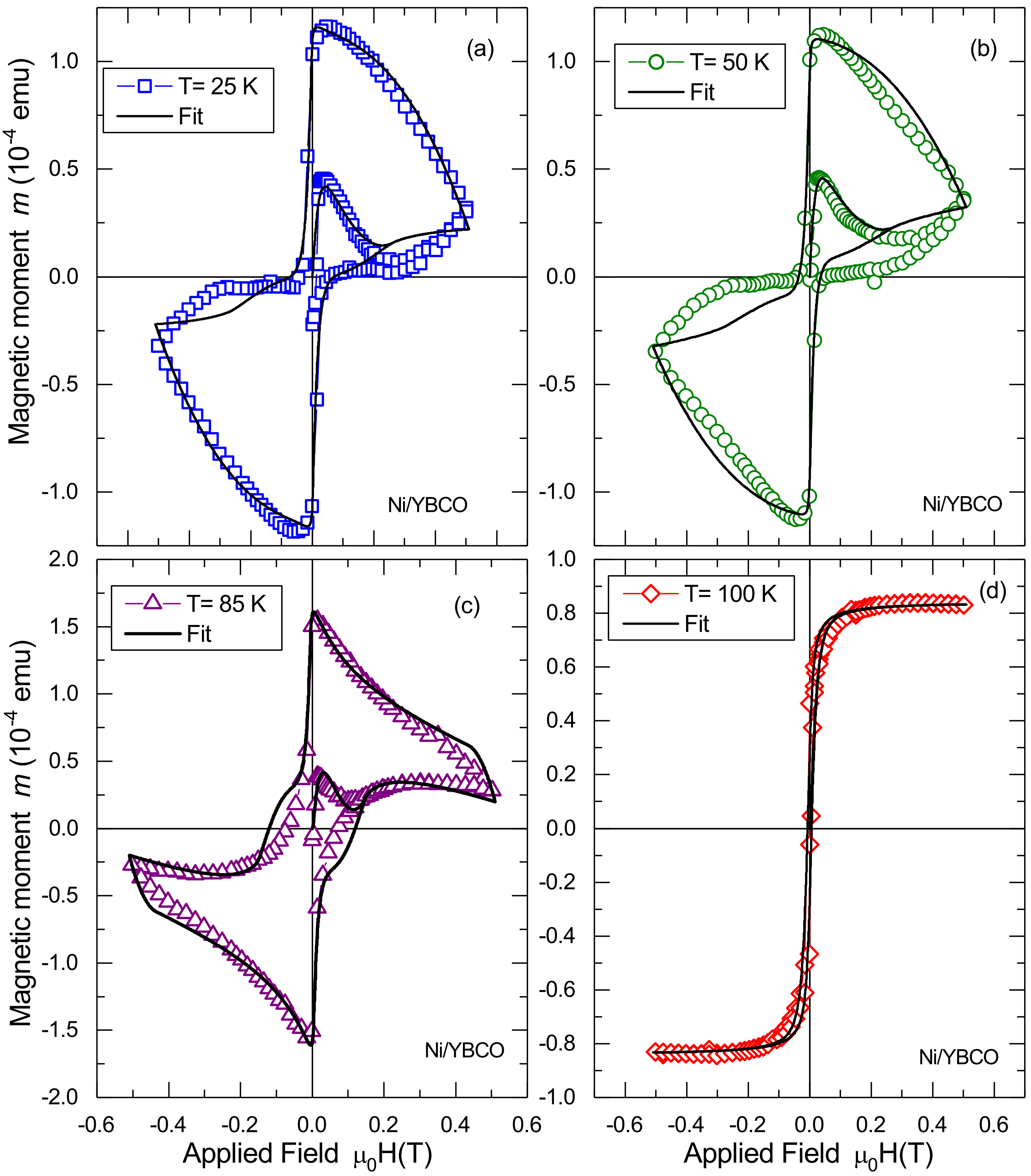

Fig. S 13. Field hysteresis loops of the Ni/YBCO bilayer film at different temperatures. From (a) to (d) the open symbols are the experimental results and the continuous lines the results of the fits.

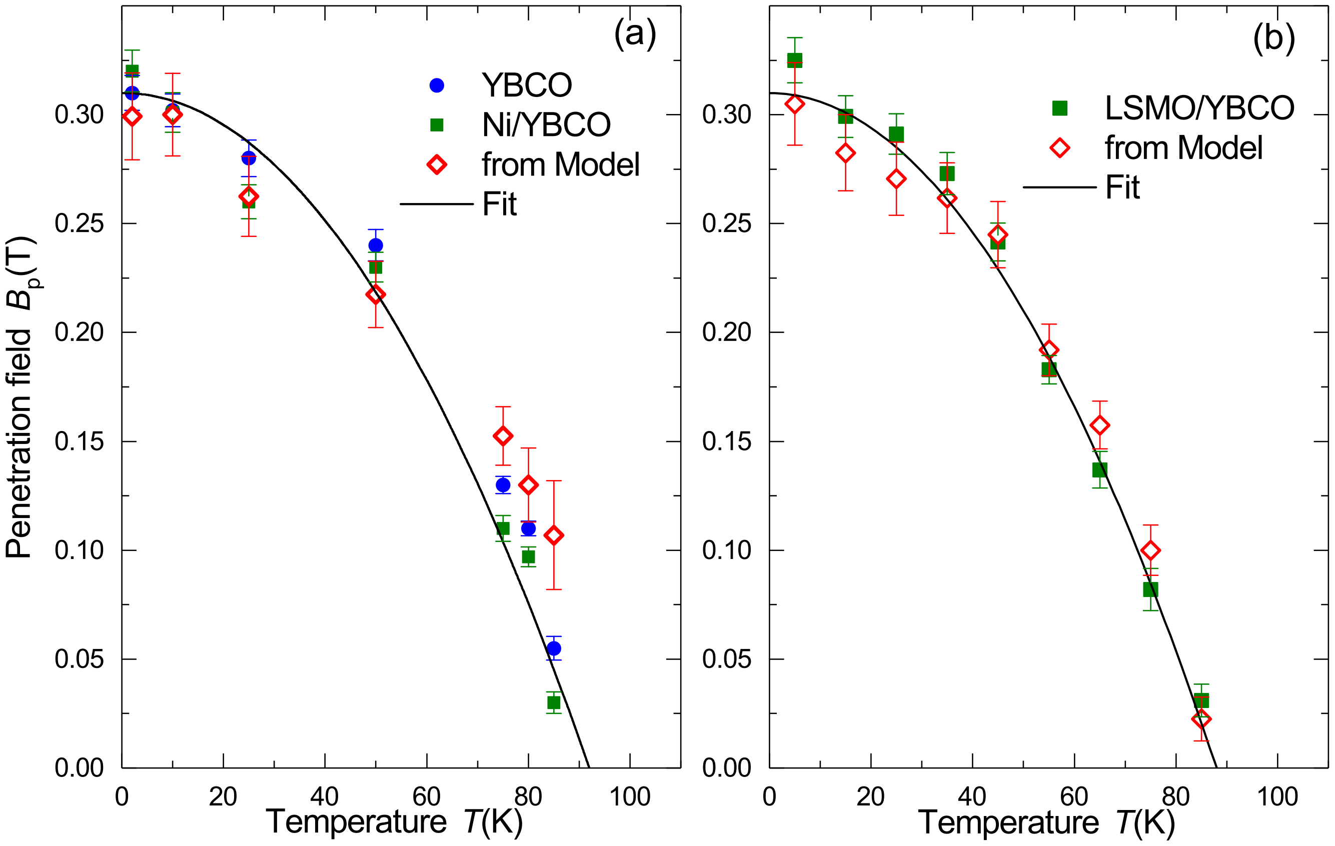

Fig. S 14. Penetration field $B_p(T) = \mu_0 H_p$ obtained from the fits (open symbols) to the hysteresis loops using Eq. (1) (see main text) compared to the experimental results (full symbols) of: (a) the YBCO film alone and Ni/YBCO bilayer and (b) the YBCO/LSMO bilayer. The lines are fits to the equation $B_p(T) = B_0(1 - (T/T_c)^2)$.

[6] S. R. Boyd, I. Kiflawi, and G. S. Woods, Phyl. Mag. B **72**, 351 (1994).
[7] F. V. Kaminsky and G. K. Khachatryan, Can. Mineral **39**, 1733 (2001).
[8] C. Bradac, T. Gaebel, and J. R. Rabeau, *Nitrogen-Vacancy Color Centers in Diamond: Properties, Synthesis, and Applications*, edited by A. R. P. Mildren and A. J. R. Rabeau (Wiley-VCH Verlag GmbH & Co. KGaA, 2013).
[9] C. M. Breeding and J. E. Shigley, Gems & Gemology **45**, 96 (2009).

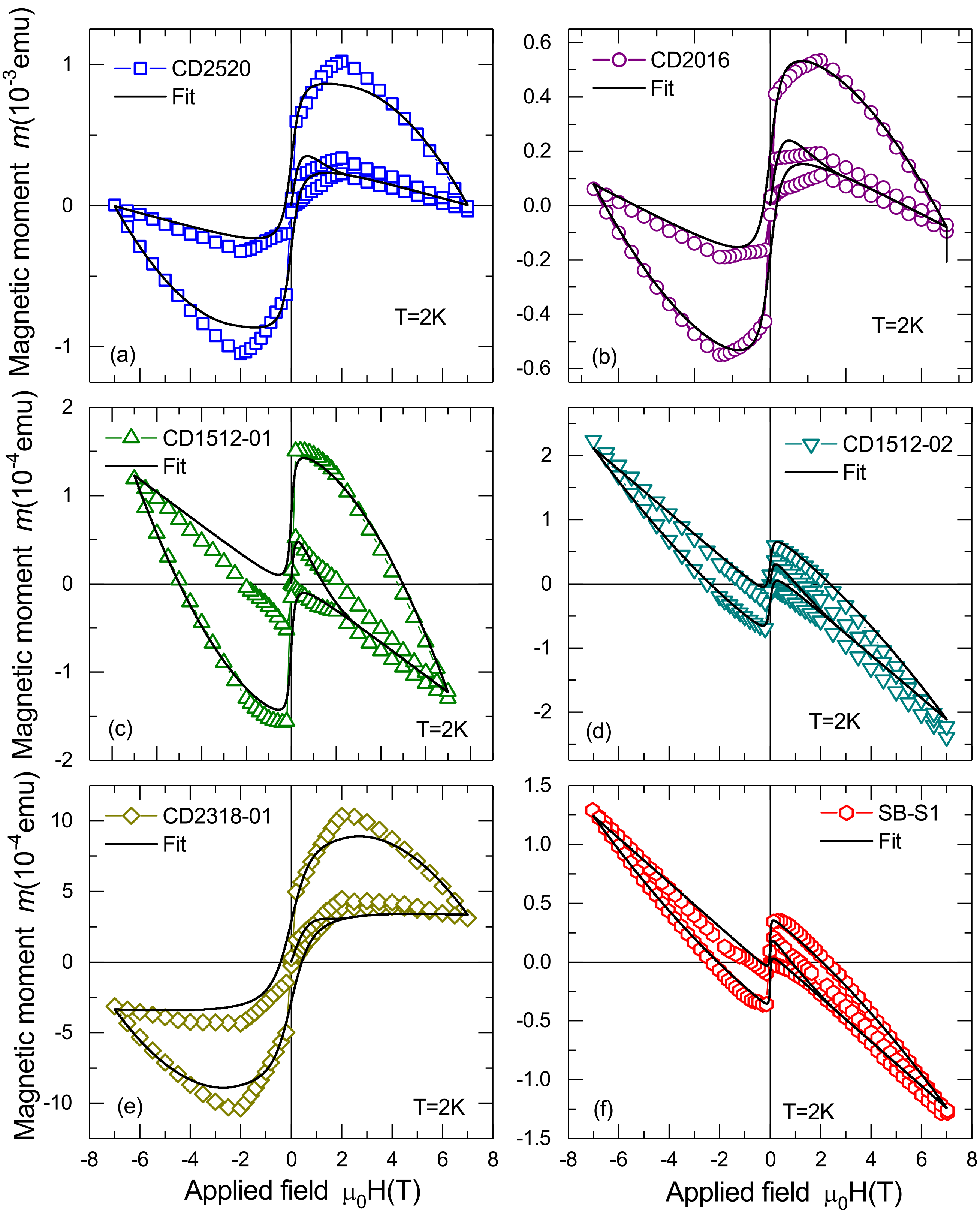

Fig. S 15. Field hysteresis loops of the N-doped diamond samples. In (a) to (f) the open symbols are the experimental results and lines the fits using Eq.(1).

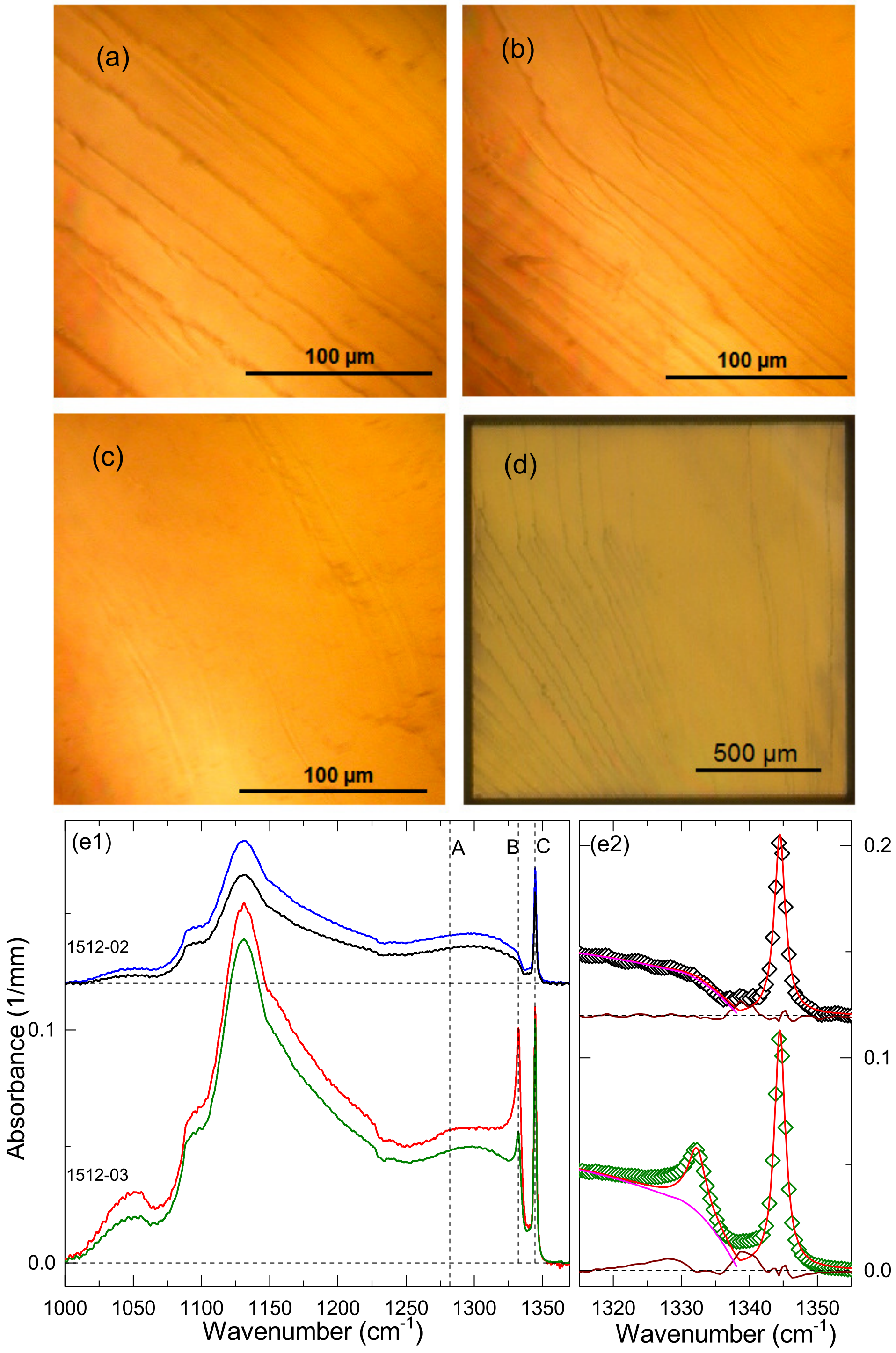

Fig. S 16. (a)-(c) Optical images of the three different regions where IR spectra were obtained, and (d) optical image of the same sample 1512-02. (e) IR-spectra and analysis: After subtraction of a straight baseline (black dashed lines) the peak at 1344 cm^{-1} (ν_C) is fitted with a Lorentzian (in red). To analyze the peak at 1335 cm^{-1} (related to B-defects), a scaled spectrum of a B-defect free sample (α_{step}: magenta) is subtracted before fitting the peak with a Lorentzian. The residuum of fit and baseline is shown as a cyan line. Spectra are shifted vertically for easy comparison. Panel (e2) is a magnification of (e1), and the spectrum of the sample 1512-02 has been scaled by a factor of 2.

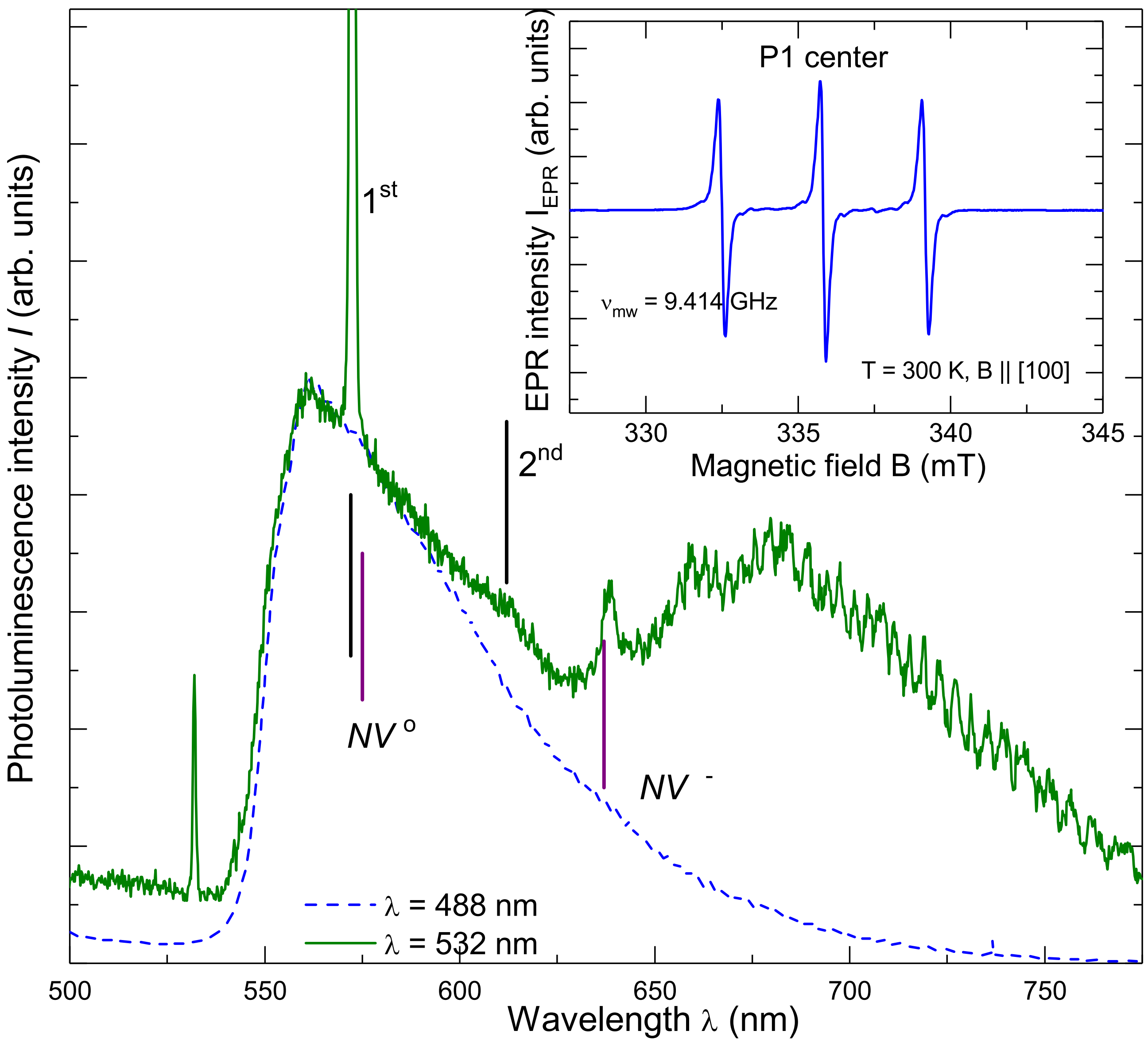

Fig. S 17. Photoluminescence spectrum measured at room temperature using two different wavelengths on sample ND-S1. The inset shows the EPR spectrum measured at room temperature that shows the existence of the so-called P1 centre due to the nitrogen doping.
